# Efficacy and Safety of Probiotics for the Treatment of Alzheimer's Disease, Mild Cognitive Impairment, and Parkinson's Disease: A Systematic Review and Meta-Analysis

**DOI:** 10.3389/fnagi.2022.730036

**Published:** 2022-02-03

**Authors:** Shuai Xiang, Jin-Long Ji, Sha Li, Xi-Peng Cao, Wei Xu, Lan Tan, Chen-Chen Tan

**Affiliations:** ^1^Department of Gastroenterology, Affiliated Hospital of Qingdao University, Qingdao, China; ^2^Department of Cardiology, Qingdao Municipal Hospital, Qingdao University, Qingdao, China; ^3^Department of Gynaecology and Obstetrics, Qingdao Women and Children's Hospital, Qingdao University, Qingdao, China; ^4^Clinical Research Center, Qingdao Municipal Hospital, Qingdao University, Qingdao, China; ^5^Department of Neurology, Qingdao Municipal Hospital, Qingdao University, Qingdao, China

**Keywords:** probiotics, Alzheimer's disease, mild cognitive impairment, Parkinson's disease, cognitive, inflammation, meta-analysis

## Abstract

**Background:**

Alzheimer's disease (AD) and Parkinson's disease (PD) are two of the most common neurodegenerative diseases, and mild cognitive impairment (MCI) is considered a prodromal stage of clinical AD. Animal studies have shown that probiotics can improve cognitive function and mitigate inflammatory response, however, results from randomized controlled trials in humans are still unclear.

**Objectives:**

A systematic review and meta-analysis was conducted to evaluate the efficacy and safety of probiotic therapy on cognitive function, oxidative stress, and gastrointestinal function in patients with AD, MCI, and PD.

**Methods:**

We searched the electronic databases such as PubMed, EMBASE, Cochrane Library until October 2020 for the eligible randomized controlled trials, as well as the unpublished and ongoing trials. Our primary endpoints were cognitive function, inflammatory and oxidative stress biomarkers, gastrointestinal function, and adverse events.

**Results:**

After screening 2,459 titles and abstracts about AD or MCI, we selected 6 eligible studies (*n* = 499 patients). After screening 1,923 titles and abstracts about PD, we selected 5 eligible studies (*n* = 342 patients). Compared with the control group, treatment with probiotics improved the cognitive function of patients with AD in the intervention group (*P* = 0.023). Cognitive function also improved in MCI patients (*P* = 0.000). Inflammation-related indicators: Malondialdehyde (MDA) was significantly reduced (*P* = 0.000); and hs-CRP decreased (*P* = 0.003). Lipid-related indicators: VLDL decreased (*P* = 0.026); triglyceride decreased (*P* = 0.009); and insulin resistance level improved: decreased Homeostatic Model Assessment for Insulin Resistance (HOMA-IR) (*P* = 0.019).

**Conclusion:**

Our analyses suggest that probiotics can improve cognitive and gastrointestinal symptoms in patients with AD, MCI, and PD, which is possibly through reducing inflammatory response and improving lipid metabolism. The safety has also been proven. However, more RCTs with rigorous study design are needed to support our findings.

**Systematic Review Registration:**

PROSPERO, Identifier: CRD42021231502.

## Introduction

Neurodegenerative diseases are a heterogeneous of disorders characterized by the progressive degeneration of the structure and function of the central nervous system (CNS) or peripheral nervous system (PNS). These age-related diseases are becoming more common, partly because the elderly population has grown in recent years (Heemels, 2016). Common neurodegenerative diseases include AD and PD (Fratiglioni and Qiu, 2009). MCI is considered a prodromal stage of clinical AD (Petersen, 2004). These diseases mainly affect aging individuals and progress steadily due to increased loss of specific neurons in the brain.

The intestinal microbiome refers to the symbiotic microorganisms such as bacteria, archaea, viruses and fungi that live in the human gut. For thousands of years, they have co-evolved with their hosts to form an intricate and mutually beneficial relationship (Guarner and Malagelada, 2003; Thursby and Juge, 2017). The gut-brain axis (GBA) is a bidirectional link between the CNS and the enteric nervous system (ENS) (Skonieczna-Zydecka et al., 2018). It involves direct and indirect pathways connecting cognitive and affective centers in the brain with peripheral intestinal function. Progressive loss of selectively vulnerable populations of neurons is a pathological manifestation of neurodegenerative diseases such as AD and PD (Dugger and Dickson, 2017). Although the etiology of neurodegenerative diseases is still unclear, numerous studies have implicated that inflammation may be involved in the process of these diseases (Dugger and Dickson, 2017). A growing recognition that the immune system is inextricably involved in shaping the brain during development, as well as mediating damage in neurodegenerative diseases, has prompted therapeutic approaches to the regulation of the immune system. But the regulatory mechanism of the immune system remains unclear (Stephenson et al., 2018). Studies have shown that in AD patients, there are intestinal microbes with decreased density and a decreased colony size, and there is a rich source of pro-inflammatory bacteria and lower anti-inflammatory bacteria (e.g., Bacillus fragilis, Eubacterium rectale, Eubacterium hallii, Faecalibacterium prausnitzii, and Bacteroides fragilis) in amyloid-positive patients as compared to healthy subjects (Kesika et al., 2021). MCI and AD patients shared similar alterations in gut microbiota (Li et al., 2019). In particular, the increase of gram-negative bacilli in patients with AD may lead to increased migration of Lipopolysaccharide (LPS) from the gut to the systemic circulation, which in turn promotes or aggravates the pathology of AD through neuroinflammation (Spielman et al., 2018). Clinical studies have shown a significant reduction in cellulose-degrading bacteria and a significant increase in hypothetical pathogens such as Escherichia coli, Streptococcus, Proteus and Enterococcus in fecal samples from PD patients compared to healthy controls (Cirstea et al., 2020). Notably, streptococci produce neurotoxins such as streptomycin and streptokinase, which can cause permanent nerve damage (Li et al., 2017). Therefore, if we can increase the number of anti-inflammatory bacteria in the gut of AD and PD patients and keep the gut bacteria stable, it may be possible to suppress inflammation and slow down the two diseases. Intestinal flora is a potential target for treatment of these diseases.

Probiotics are living microbes that are good for health when consumed in regular amounts, which may be explained by their anti-inflammatory or antioxidant properties (Lynch and Pedersen, 2016; Wallace and Milev, 2017; Chunchai et al., 2018). In recent years, several studies have found the anti-inflammatory effects of probiotics. A systematic review of 11 animal studies by Wang et al. showed that almost all the studies found significant effects on measured CNS functions, except for one testing effect of Bifidobacterium infantis on depression-like behavior (Wang et al., 2016). These preclinical results suggested that probiotics might be an effective dietary intervention to ameliorate age-related cognitive deficits. However, the results of previous clinical trials on the effects of probiotics on patients with PD, AD, or MCI were inconsistent (Akbari et al., 2016; Barichella et al., 2016; Georgescu et al., 2016; Agahi et al., 2018; Borzabadi et al., 2018; Hwang et al., 2019; Kobayashi et al., 2019; Tamtaji et al., 2019a,b; Xiao et al., 2020; Tan et al., 2021). Therefore, it is necessary to conduct a meta-analysis and systematic review of these RCT trials to further explore the effects of probiotics on various biochemical indicators and cognitive function, as well as explore the potential mechanisms.

## Materials and Methods

### Literature Search

This meta-analysis complies with the systematic review and meta-analysis report program recommended by the PRISMA guidelines. The preliminary search was performed in October 2020 through the PubMed, Embase, and Cochrane Library without time restrictions. We searched the following terms in “all fields” in every electronic database: (probiotic OR yeast OR yogurt OR fermented product OR lactobacillus OR bifidobacterium OR fermented dairy product OR synbiotics OR cultured milk products) AND (Alzheimer's disease OR dementia OR mild cognitive impairment OR cognitive dysfunction OR cognitive defect OR cognition OR memory OR mental capacity); (probiotic OR yeast OR yogurt OR fermented product OR lactobacillus OR bifidobacterium OR fermented dairy product OR synbiotics OR cultured milk products) AND (Parkinson OR Parkinson's disease OR parkinsonism). Articles were limited to randomized controlled trials (RCTs) in humans. The references of relevant articles were also checked to identify additional eligible studies. The titles and abstracts of articles were initially and independently screened for eligibility by two investigators. Duplicate and irrelevant papers were excluded. For the relevant candidates, the full articles were retrieved for review. And the references of each document were checked to identify potential candidates. Disagreements were resolved through discussion between the two researchers or with a third reviewer.

### Inclusion and Exclusion Criteria

Included studies had to meet the following criteria: (1) The study was a randomized controlled trial (RCT); (2) Adult human participants who had a diagnosis of AD, MCI, or PD (aged over 18 y). Mild cognitive impairment (MCI) refers to a state of cognitive deterioration that precedes the clinical diagnosis of Alzheimer's disease (AD) and other dementias, which does not yet compromise daily functioning; (3) Pattern, taste, and smell of probiotics and placebo shouldn't have any significant difference at baseline; (4) Full English text; (5) Continuous data at baseline and post-intervention, or the change from baseline, and the number of participants at baseline and post-intervention were reported or could be calculated from the data reported in the article. Studies were excluded if they met any one of the following criteria: (1) Case reports or case series; (2) Abstracts, comments, reviews, letters, and conference speeches; (3) Nonhuman (*in vitro* and animal) research; (4) Changes in study indicators relative to baseline intervention were not reported, or information-based data could not be calculated. (5) The study reported on a sample that overlapped the sample in another study. In this case, only the study with the larger sample size was included.

### Data Extraction

Data were extracted independently by two investigators using a predetermined format in accordance with the guidance of the Cochrane Handbook for Systematic Reviews of Interventions. Basic information of the RCTs was extracted from the included studies: (1) Name of the first author; (2) Date of publication; (3) Sample size; (4) Age and sex; (5) Species of strain, duration and dose; (6) Primary and secondary results; (7) Primary findings. For missing data, we sought missing information and essential clarification from the author. To perform the meta-analysis, we extracted the mean change score (standard deviation [SD] or standard error of the mean [SEM]) of the included variables. When change scores were not available, the scores (mean ± SD or mean ± SEM) and the numbers of participants at baseline and post-intervention were extracted.

### Statistical Analysis

The primary outcomes of this study were the standardized mean differences (SMDs) of changes in MMSE, TYM, and RBANS from baseline between experimental group and control group. If the baseline standard deviation is not available, we calculated it using confidence interval (CI), standard error (SE), and T values according to the principles in the Cochrane Manual. The SMD was tested by a Z statistic, and a two-tailed *P* < 0.05 was regarded as statistically significant. The interstudy heterogeneity was examined by chi-square (χ^2^) statistics and *I*^2^ statistics. The heterogeneity among the different studies was considered high if *P* < 0.1 for the χ^2^ statistic or *I*^2^ > 50% (Cumpston et al., 2019). SMDs were calculated by fixed-effects or random-effects models. A sensitivity analysis was conducted to test the reliability of the findings using the leave-one-out method, and the publication bias was assessed by Egger's test and Begg's test. Forest plots, sensitivity analysis, Egger's test, and Begg's test were performed in STATA16 software, and Revman 5.3. was used to generate the summary of risk of bias assessment.

## Results

### Literature Search and Screening

**Figure 1:**
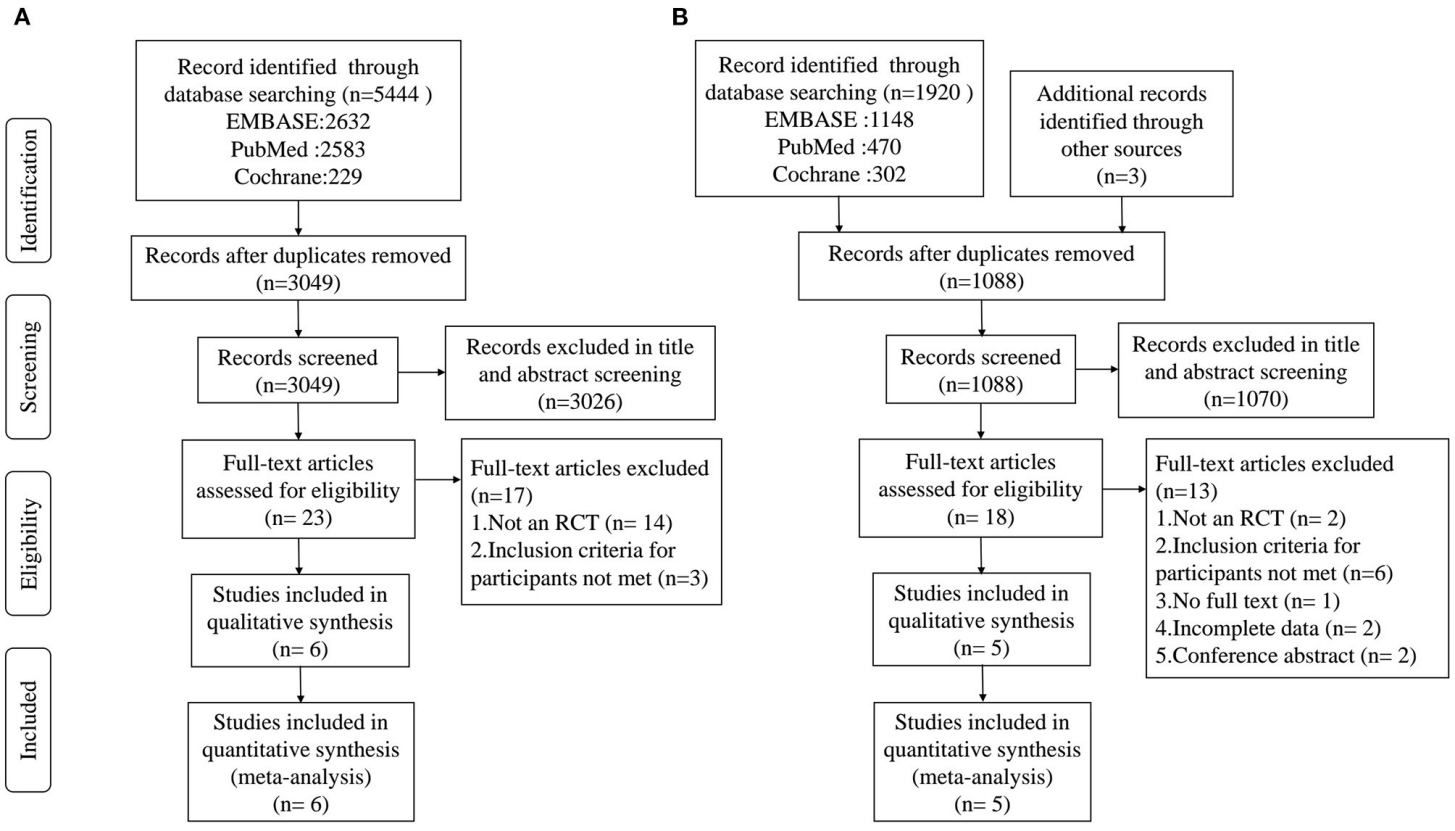
PRISMA flow diagram of the literature search and abstraction process. **(A)** AD and MCI; **(B)** PD.

For AD or MCI, a total of 5,444 records were obtained after the initial search of the electronic databases. Of these, 2,395 trials were removed as duplicates, and 3,026 publications were excluded after screening the titles and abstracts. The remaining 23 articles were scrutinized by full text, of which 17 were excluded for reasons detailed in the PRISMA flow chart (Figure 1). Then, 6 studies were considered eligible and were eventually included in the quantitative meta-analysis. For PD, 1,920 articles were obtained after the initial search of the electronic databases and 3 studies were identified through a manual search of the reference lists of relevant published reviews. Of these, 832 trials were removed as duplicates, and 1,070 publications were excluded after screening the titles and abstracts. The remaining 18 articles were scrutinized by full text, of which 13 were excluded for reasons detailed in the PRISMA flow chart (Figure 1). Then, 5 qualified articles were included in the quantitative meta-analysis.

### Study Characteristics

As shown in Table 1, the publication years of the 6 included studies on AD or MCI patients ranged from 2016 to 2020, with an aggregated sample of 499 individuals. All the 6 studies were randomized, double-blind, controlled trials, among which three studies (Akbari et al., 2016; Agahi et al., 2018; Tamtaji et al., 2019a) recruited subjects diagnosed with AD and the other three (Hwang et al., 2019; Kobayashi et al., 2019; Xiao et al., 2020) included people with mild cognitive impairment. Three studies had higher proportions of women; two (Kobayashi et al., 2019; Xiao et al., 2020) had balanced proportions; and the last one (Tamtaji et al., 2019a) did not report the sex ratio of the recruited subjects. The intervention duration of most included studies was 12 weeks except for one study (Xiao et al., 2020) whose duration was 16 weeks. Three studies recruited subjects diagnosed with AD using multiple strains of probiotics, and the other three included patients with MCI using a sole strain. All studies used probiotic-matched placebos which were indistinguishable from the probiotics in terms of packaging, shape, appearance, size, taste, smell, and so on. Of the three AD studies, two (Akbari et al., 2016; Tamtaji et al., 2019a) found significant improvements in cognition in the probiotics group compared with the control group, while the third (Agahi et al., 2018) did not. One of the studies (Tamtaji et al., 2019a) divided subjects into three groups: a control group, a selenium group, and a probiotic-selenium group. In order to explore the influence of probiotics, we only included the experimental data of the latter two groups for the study. Of the three MCI studies, two (Hwang et al., 2019; Xiao et al., 2020) found significant differences, and one (Kobayashi et al., 2019) reported mixed findings. Similarly, the results of studies about the influences of probiotics on various inflammatory and oxidative metabolites have been inconsistent.

**Table 1:** Main characteristics of the included AD and MCI studies.

**References**	**Study design**	***N***	**Diagnostic criteria**	**Age (M** **±** **SD)**		**Sex ratio (M/F)**		**Type of Probiotics**	**Duration (weeks)**	**Dose**	**Primary outcome**	**Secondary outcome**
				**PRO**	**CON**	**PRO**	**CON**					
Akbari et al. (2016)	Randomized Double-blind Placebo-Controlled Trial	60	AD(NINDS-ADRDA criteria)	77.67± 2.62	82.00 ± 1.69	6/24	6/24	Multiple (Lactobacillus acidophilus, Lactobacillus casei, Bifidobacterium bifidum, Lactobacillus fermentum)	12	8 × 10^9^ (CFU/g)	MMSE	TAC GSH MDA hs-CRP NO
Agahi et al. (2018)	Randomized Double-blind Placebo-Controlled Trial	48	AD (NINDS-ADRDA criteria)	79.70 ± 1.72	80.57 ± 1.79	7/18	10/13	Multiple (*Lactobacillus fermentum, Lactobacillus plantarum, Bifidobacterium lactis Lactobacillus acidophilus, Bifidobacterium bifidum, Bifidobacterium longum*)	12	3 × 10^9^ (CFU/d)	TYM	TAC GSH MDA NO
Tamtaji et al. (2019a)	Randomized Double-blind Placebo-Controlled Trial	90	AD (NINDS-ADRDA criteria)	76.2 ± 8.1	78.5 ± 8.0	/	/	Multiple (Lactobacillus acidophilus, Bifidobacterium bifidum, Bifidobacterium longum)	12	6 × 10^9^ (CFU/d)	MMSE	TAC GSH MDA hs-CRP NO
Kobayashi et al. (2019)	Randomized Double-blind Placebo-Controlled Trial	121	Subjective memory complaints (MMSE, 22–27)	61.5 ± 6.83	61.6 ± 6.37	30/31	30/30	Sole (*Bifidobacterium breve* A1)	12	>2.0 × 10^10^ (CFU/d)	RBANS MMSE	hs-CRP
		44	MCI (RBANS <41)					Sole (*Bifidobacterium breve* A1)	12	>2.0 × 10^10^ (CFU/d)	RBANS MMSE	
Hwang et al. (2019)	Randomized Double-blind Placebo-Controlled Trial	100	MCI (DSM-5)	68.0 ±5.12	69.2 ± 7.00	20/30	14/36	Sole (*Lactobacillus plantarum* C29)	12	>1.0 × 10^10^ (CFU/d)	VLT ACPT DST	
Xiao et al. (2020)	Randomized Double-blind Placebo-Controlled Trial	80	MCI (MMSE ≥22)	61.3 ± 7.7	60.9 ± 6.9	19/21	20/20	Sole (*Bifidobacterium breve*A1)	16	>2.0 × 10^10^ (CFU/d)	RBANS	JMCIS

*PRO, probiotics group; CON, control group; AD, Alzheimer's disease; MCI, mild cognitive impairment; NINCDS-ADRDA, National Institute of Neurological and Communicative Diseases and Stroke/Alzheimer's Disease and Related Disorders Association; DSM-5, Diagnostic and Statistical Manual of Mental Disorders, 5th edition; CFU, colony-forming units; MMSE, Mini-Mental State Examination; TAC, total anti-oxidant capacity; GSH, total glutathione; MDA, malondialdehyde; hs-CRP, high-sensitivity C-reactive protein; NO, nitric oxide; TYM, test your memory; RBANS, Repeatable Battery for the Assessment of Neuropsychological Status; VLT, verbal learning test; ACPT, auditory continuous performance test; DST, digit span test; JMCIS, Japanese version of the MCI Screen*.

As shown in Table 2, five PD studies were published from 2016 to 2020 with a total sample size of 342 people. All these studies were randomized, double-blind, controlled trials. One (Georgescu et al., 2016) was based on the modified Hoehn-Yars scale; another (Tan et al., 2021) was based on the Queen Square Brain Bank; and the remaining three (Barichella et al., 2016; Borzabadi et al., 2018; Tamtaji et al., 2019b) were based on the UK Brain Bank. All but two studies (Georgescu et al., 2016; Tamtaji et al., 2019b) reported sex ratios, in which the participants included were mostly male. Multiple strains were used in all the studies. The intervention duration of two included studies (Barichella et al., 2016; Tan et al., 2021) was 4 weeks, and that of the other three (Georgescu et al., 2016; Borzabadi et al., 2018; Tamtaji et al., 2019b) was 12 weeks. All of the studies used probiotic-matched placebos which were indistinguishable from the probiotics in terms of packaging, shape, appearance, size, taste, smell, and so on. Regarding the main findings, three studies (Barichella et al., 2016; Georgescu et al., 2016; Tan et al., 2021) reported that the probiotics intervention group significantly improved the gastrointestinal symptoms of PD patients, such as abdominal pain, abdominal distension, constipation, and other gastrointestinal symptoms. Besides, two studies (Borzabadi et al., 2018; Tamtaji et al., 2019b) found that the probiotics intervention group reduced the gene expression of some inflammatory markers and improved cognition.

**Table 2:** Main characteristics of the included PD studies.

**Study**	**Study design**	***N***	**Diagnostic criteria**	**Age (M** **±** **SD)**		**Sex ratio (M/F)**		**Type of Probiotics**	**Duration (weeks)**	**Dose**	**Primary outcome**	**Secondary outcome**	**Main findings**
				**PRO**	**CON**	**PRO**	**CON**						
Georgescu et al. (2016)	Randomized Double-blind Placebo- Controlled Trial	40	PD (modified Hoehn–Yars scale)	69.80 ± 5.64	75.65 ± 9.66	/	/	Multiple (*Lactobacillus acidophilus, Bifidobacterium infantis*)	12	120 mg/d	Abdominal pain; Bloating; Constipation	Non-motor symptoms	Treatment with probiotics could improve abdominal pain and bloating as much as with trimebutine, but less for constipation with incomplete evacuation, where trimebutine showed better results.
Barichella et al. (2016)	Randomized Double-blind Placebo- Controlled Trial	120	PD (UK Brain Bank criteria and Rome III criteria)	71.8 ± 7.7	69.5 ± 10.3	41/39	24/16	Multiple (*Streptococcus salivarius subsp thermophilus, Enterococcus faecium, Lactobacillus rhamnosus, Lactobacillus acidophilus, Lactobacillus plantarum*)	4	2.5 × 10^11^ (CFU/d)	CBMs	3 or more CBMs; CBMs during weeks 3 and 4; stool frequency; stool consistency; the frequency of laxative use; satisfaction with treatment	The consumption of a fermented milk containing multiple probiotic strains and prebiotic fiber was superior to placebo in improving constipation in patients with PD.
Borzabadi et al. (2018)	Randomized Double-blind Placebo- Controlled Trial	50	PD (the UK PD Society Brain Bank criteria)	66.9 ± 7.0	66.7 ± 10.7	17/8	16/9	Multiple (*Lactobacillus acidophilus, Bifidobacterium bifidum, L. reuteri, Lactobacillus fermentum*)	12	8 × 10^9^ (CFU/d)	IL-1 IL-8 TNF-α TGF-β VEGF PPAR-γ	NO GSH GAPDH LDLR	Probiotics supplementation for 12 weeks in PD patients significantly improved gene expression of IL-1, IL-8, TNF-α, TGF-β, and PPAR-γ, but did not affect gene expression of VEGF and LDLR, and biomarkers of inflammation and oxidative stress.
Tamtaji et al. (2019b)	Randomized Double-blind Placebo- Controlled Trial	60	PD (the UK PD Society Brain Bank clinical diagnostic criteria)	68.2 ± 7.8	67.7 ± 10.2	/	/	Multiple (*Lactobacillus acidophilus, Bifidobacterium bifidum, Lactobacillus reuteri, Lactobacillus fermentum*)	12	8 × 10^9^ (CFU/d)	MDS-UPDRS hs-CRP	TAC GSH MDA FPG LDL HDL VLDL HOMA-IR QUICKI Insulin Triglycerides Total cholesterol	Our study evidenced that 12 weeks of probiotic consumption by individuals with PD had useful impacts on MDS-UPDRS and few metabolic profiles
Tan et al. (2021)	Randomized Double-blind Placebo- Controlled Trial	72	PD (Queen Square Brain Bank Criteria and Rome IV criteria)	70.9 ± 6.6	68.6 ± 6.7	20/14	28/10	Multiple (*E. faecium, L. acidophilus, L. paracasei, L. rhamnosus, B. longum, B. bifidum, L. reuteri*)	4	1 × 10^10^ (CFU/d)	SBMs	Stool consistency; PAC-QOL; constipation severity score; laxative usage; satisfaction with treatment	SBM and secondary outcomes including stool consistency and quality of life related to constipation increased after treatment with probiotics.

*PRO, probiotics group; CON, control group; PD, Parkinson's disease; CFU, colony-forming units; CBM, complete bowel movement; IL-1, Interleukin-1; IL-8, Interleukin-1; TNF-α, tumor necrosis factor-α; TGF-β, transforming growth factor-β; VEGF, vascular endothelial growth factor; PPAR-γ, peroxisome proliferator activated receptor gamma; NO, nitric oxide; GSH, total glutathione; GAPDH, glyceraldehyde-3-Phosphate dehydrogenase; LDLR, low-density lipoprotein receptor; MDS-UPDRS, Movement Disorders Society-Unified Parkinson's Disease Rating Scale; hs-CRP, high-sensitivity C-reactive protein; TAC, total anti-oxidant capacity; GSH, total glutathione; MDA, malondialdehyde; FPG, fasting plasma glucose; LDL, low density lipoprotein; HDL, high density lipoprotein; VLDL, very low density lipoprotein; HOMA-IR, Homeostatic Model Assessment for Insulin Resistance; QUICKI, quantitative insulin-sensitivity check index; SBM, spontaneous bowel movements; PAC-QOL, Patient Assessment of Constipation Quality of Life*.

### Risk of Bias Assessment

**Figure 2:**
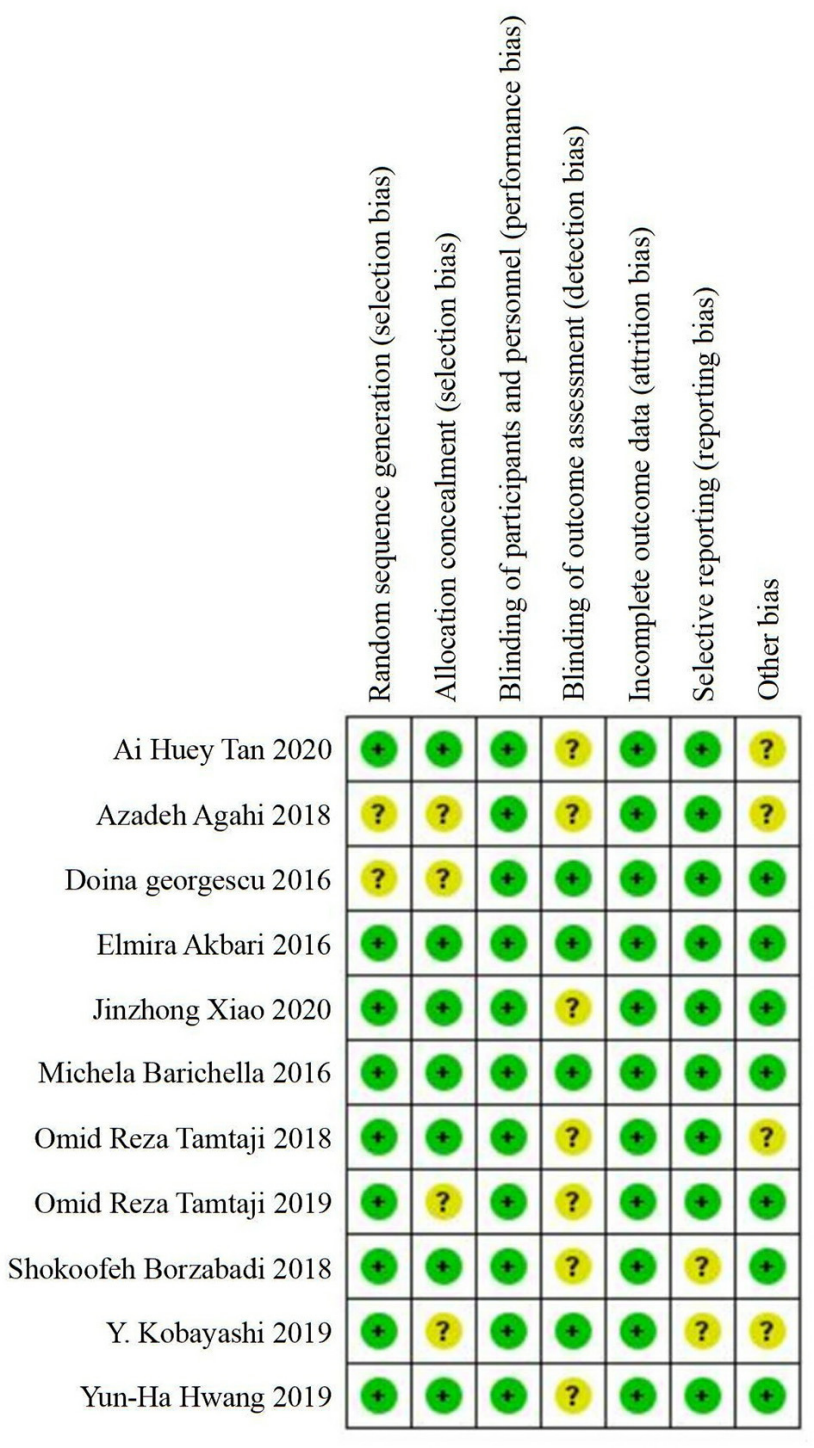
Summary of risk of bias assessment: judgements of the review authors on each risk of bias item for the included studies (*n* = 11).

A total of 11 included studies were randomized controlled trials, but two studies (Georgescu et al., 2016; Agahi et al., 2018) did not provide information about random sequence generation and four studies (Georgescu et al., 2016; Agahi et al., 2018; Kobayashi et al., 2019; Tamtaji et al., 2019a) did not provide information about allocation concealment. All of the studies described the blindness of the participants and personnel, whereas only four studies (Akbari et al., 2016; Barichella et al., 2016; Georgescu et al., 2016; Kobayashi et al., 2019) reported the blindness of outcome assessments. No risk of incomplete data was found in any of the included studies. In general, the assessment of bias reported a low to moderate risk of bias across all areas. Figure 2 summarizes the risks of bias assessment across the recruited studies.

### Meta-Analysis: Main Results

Three AD studies included 85 patients in the probiotics group and 83 patients in the control group. In these three studies, due to acceptable heterogeneity, fixed effect models were selected for quantitative synthesis, and Mini-mental State Examination (MMSE) scores reflecting cognition were significantly different between the probiotics group and the control group (SMD = 0.36; 95% CI, 0.05–0.68; *P* = 0.023; *I*^2^ = 52.0%). In addition, there were significant differences in inflammation-related indicators: hs-CRP (SMD = −0.57; 95% CI, −0.95 to −0.2; *P* = 0.003; *I*^2^ = 0.0%), MDA (SMD = −0.57; 95% CI, −0.89 to −0.26; *P* = 0.000; *I*^2^ = 0.0%), and HOMA-IR (SMD = −0.45; 95% CI, −0.82 to −0.07; *P* = 0.019; *I*^2^ = 0.0%). Lipid-related indicators: VLDL (SMD = −0.42; 95% CI, −0.8 to −0.05; *P* = 0.026; *I*^2^ = 0.0%) and triglyceride (SMD = −0.5; 95% CI, −0.88 to −0.13; *P* = 0.009; *I*^2^ = 0.0%).

**Figure 3:**
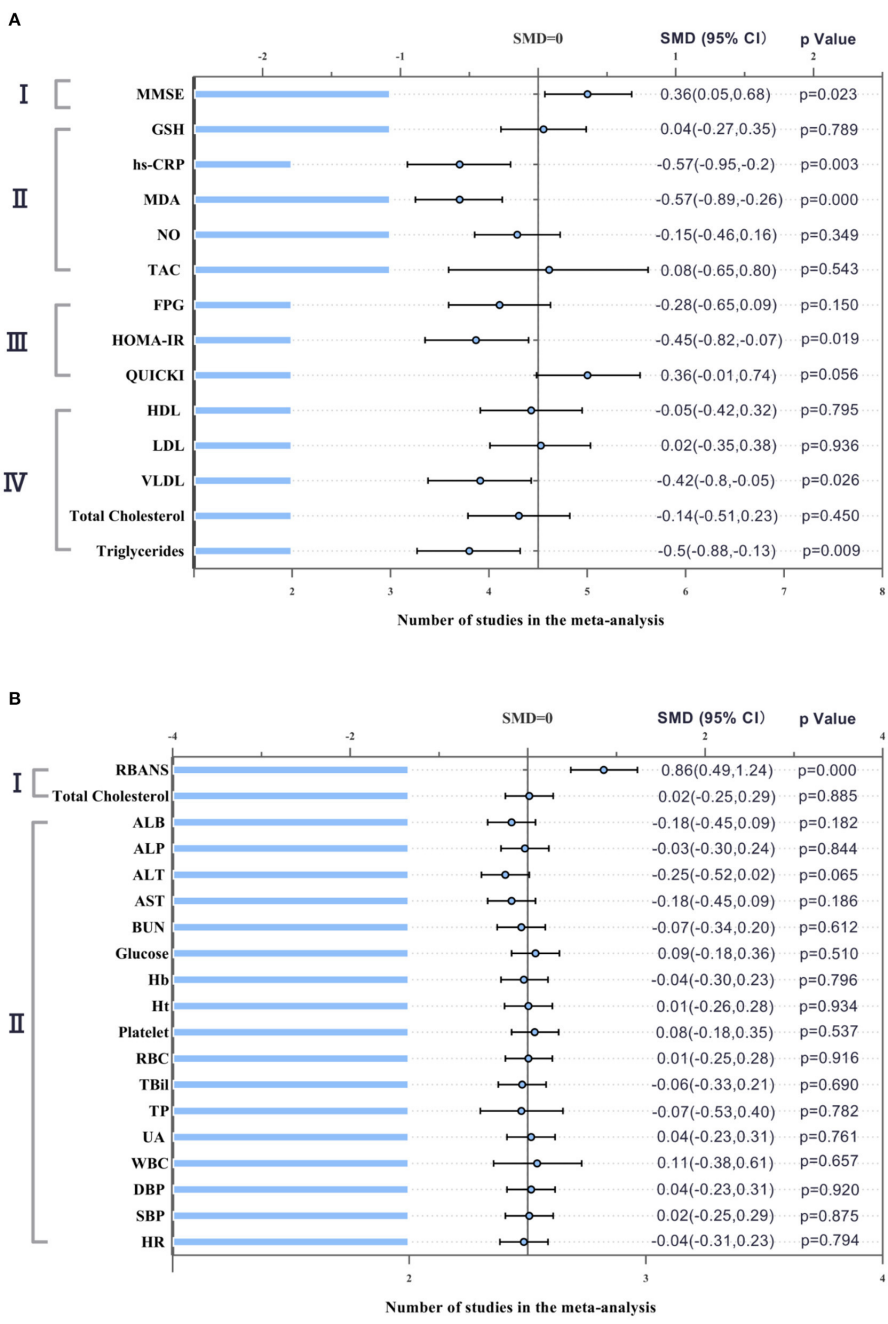
Forest plot showing the effects in the probiotics group versus the control group on cognitive function and biochemical indicators. **(A)** Meta-analysis of the effects of probiotics on patients with AD. I. MMSE (Mini-Mental State Examination); II. Biomarkers of inflammation and oxidative stress. GSH (glutathione); hs-CRP (hypersensitive-CRP); MDA (Malondialdehyde); NO (Nitric Oxide); TAC (tricarboxylic acid cycle); III. Glucose related indicators. FPG (fasting plasma glucose); HOMA-IR (Homeostasis model assessment); QUICKI (Quantitative insulin sensitivity check index); IV. Lipid related indicators. HDL (high-density lipoprotein); LDL (low-density lipoprotein); VLDL (very low density lipoprotein). **(B)** Meta-analysis of the effects of probiotics on patients with MCI. I. RBANS (Repeatable Battery for the Assessment of Neuropsychological Status); II. Hematological and biological blood parameters and vital signs. ALB (Albumin); ALP (Alkaline phosphatase); ALT (Alanine transaminase); AST (Aspartate aminotransferase); BUN (Blood Urea Nitrogen); Hb (Hemoglobin); Ht (Hematocrit); RBC (red blood cell); TBil (total bilirubin); TP (total protein); UA (Uric acid); WBC (white blood cell); DBP (diastolic blood pressure); SBP (systolic blood pressure); HR (heart rate). The differences were significant if *P*-value was <0.05.

The three MCI studies had 117 participants in the probiotics group and 107 in the control group. The fixed effect models were selected for quantitative synthesis. There was a statistically significant difference between the experimental group and the control group in cognition (SMD = 0.86; 95% CI, 0.49–1.24; *P* = 0.000; *I*^2^ = 0.0%). However, there was no statistically significant difference in total cholesterol score (SMD = 0.02; 95% CI, −0.25 to 0.29; *P* = 0.885; *I*^2^ = 0.0%). Other hematological indicators, vital signs and blood biochemical indicators were not significantly different between the two groups, suggesting that probiotics were safe for the subjects. The forest plot of the meta-analysis is shown in Figure 3.

**Figure 4:**
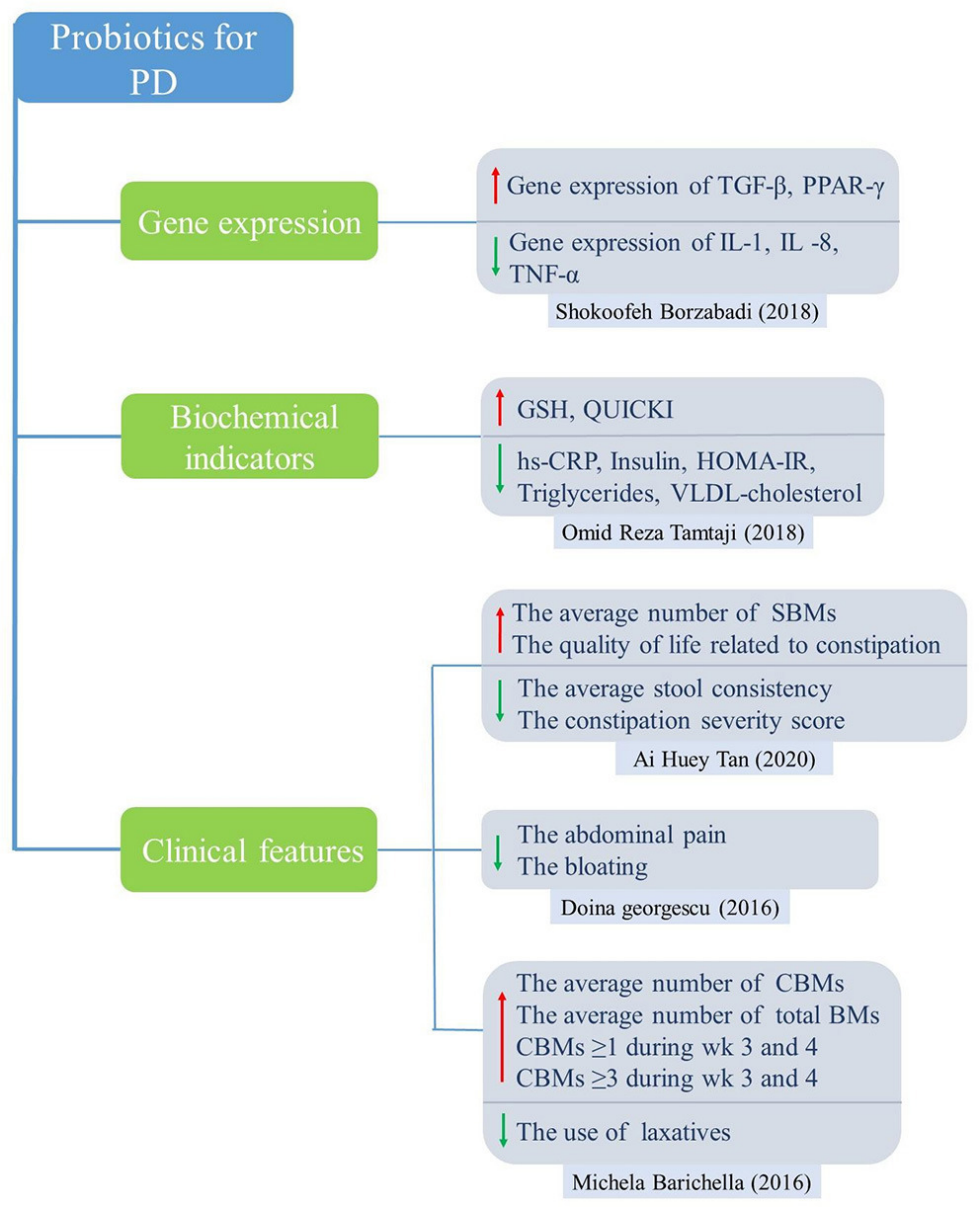
Main findings of the effects of probiotics on patients with PD.

Five PD studies included 189 subjects in the intervention group and 153 subjects in the control group. We selected appropriate effect models to synthesize the quantitative data according to the magnitude of heterogeneity. Meta-analysis results revealed a significant difference in glutathione (GSH) between the probiotics group and the control group (SMD = 0.76; 95% CI, 0.37–1.15; *P* = 0.001; *I*^2^ = 0.0%). There was no significant difference in fecal viscosity (SMD = −0.07; 95% CI, −1.32–1.18; *P* = 0.702; *I*^2^ = 94.0%). Figure 4 shows the remaining results that cannot be merged.

### Assessment of Publication Bias and Sensitivity Analysis

We conducted quantitative evaluation of publication bias by Egger's test and Begg's test. The analysis of the cognitive function among AD patients showed no significant publication bias (Egger's test: *P* = 0.993, Begg's test: *P* = 1.000). Sensitivity analysis examined the reliability of the results of the meta-analysis by omitting each study in turn. The systematic removal of each trial did not significantly affect the overall effect of probiotics on the cognition of patients with AD (see Supplementary Materials). Therefore, the findings on improved cognitive function in AD patients between the probiotics and the control group were considered reliable.

## Discussion

Neurodegenerative diseases are increasingly serious health problems among the aging population. The most common neurodegenerative diseases are AD and PD, the incidences of which are also increasing year by year. Without effective prevention and treatment, the two diseases will impose an increasing socio-economic burden. Although these two diseases have different clinical manifestations, they share similar underlying mechanisms and both associate with normal aging. Age-related changes in the brain can be observed decades before neurological function begins to decline, and studies have repeatedly linked them to immune system activation (Bangen et al., 2017). Neurodegenerative diseases, such as AD and PD, are characterized by the gradual accumulation of abnormal proteins in the CNS (Jucker and Walker, 2018). Two thousand years ago, Hippocrates declared that “all disease begins in the gut,” an interesting observation that influenced medical researchers (Cryan et al., 2019). At present, the influence of brain-gut axis on neurodegenerative diseases has attracted increasing attention. As a part of the brain-gut axis, gastrointestinal microbiota influences immunity, inflammation, and neuroregulation through the brain-gut axis. Therefore, maintaining a healthy and stable microbiome brings great benefits for immune defense, brain function, and dynamic balance (Westfall et al., 2017).

In the past decades, researchers have paid attention to the medication for AD and PD. Recently, aducanumab that targets the neurobiology of AD is approved for clinical use (Cummings and Salloway, 2021). However, the reported clinical benefits are limited and may not be apparent to individuals. In addition, the treatment has potential side effects, especially ARIA, which requires MRI monitoring for detection (Sperling et al., 2011). More recently, a growing number of research studies have shown that lifestyle changes, such as changes in diet, can ease cognitive decline (Morris et al., 2015). Probiotic intervention may be an effective measure to ameliorate age-related neurodegenerative diseases (Alkasir et al., 2017). Remarkably, our results suggest that low-cost, available, and safe probiotics could be potential candidates for the treatment of AD, MCI and PD, although the exact mechanisms remain unclear.

Oxidative stress and inflammation are two major causes of neurodegenerative diseases, and they get worse with age. Inflammation is an important risk factor for both morbidity and mortality in older adults, as most age-related diseases share the same inflammatory pathogenesis (Franceschi and Campisi, 2014). The mitochondria of cells constantly produce reactive oxygen species (ROS). In normal cells, the ROS generated can be neutralized by the antioxidant system without causing damage. However, with the aging of the organism, the defense function of cells is weakened and the cell membranes are damaged, which eventually leads to cell death (Mitsuma et al., 2008). The brain is particularly vulnerable to oxidative stress due to high oxygen consumption rates (OCRs), high polyunsaturated fatty acids, high iron content, and relatively low antioxidant capacity (Noseworthy and Bray, 1998), especially in the amygdala and hippocampal neurons (Wadhwa et al., 2018). The slow accumulation of ROS in neurons stimulates the release of cytokines, which in turn stimulates microglia activation and neuroinflammation.

For PD studies, the results of meta-analysis showed significantly higher levels of GSH in the probiotic group compared to the control group, which acts as an antioxidant, a free radical scavenger and a detoxifying agent. In addition, probiotic intake downregulated gene expression of interleukin-1 (IL-1), IL-8 and tumor necrosis factor alpha (TNF-α) but upregulated transforming growth factor beta (TGF-β) and peroxisome proliferator-activated receptor gamma (PPAR-γ). Probiotics can also improve abdominal symptoms, such as relieving abdominal pain, bloating and constipation, and increasing the number of SBMs and CBMs. For AD and MCI studies, the results showed that probiotics significantly improved cognitive function, insulin resistance, and lipid metabolism, and significantly reduced inflammatory markers such as hs-CRP and MDA.

In the elderly, overstimulation of the immune system leads to a chronic low-grade inflammatory state, which may be related to a persistent inflammatory state of the gut microbiota characterized by reduced diversity and stability (Frasca and Blomberg, 2016). In the aging process, the number of pathogens increases, such as Enterobacteriaceae, which was positively correlated with difficulty in maintaining posture and balance (Scheperjans et al., 2015). However, the probiotics and neuroprotective molecules decrease (Lambert et al., 2009; Caracciolo et al., 2014). A study involving 72 PD patients and 72 healthy subjects found that the number of Prevotellaceae in the intestines of PD patients decreased by 77.6%. Mucin produced by this microbe can form a barrier along the intestinal wall to protect against pathogen invasion. In addition, a decrease in bacteria capable of producing short-chain fatty acids (SCFA) was found in PD patients (Unger et al., 2016). The decrease in SCFA can lead to decreased expression of endothelial tight junction proteins, especially Occludin and Claudin-5, thus increasing blood-brain barrier (BBB) permeability (Braniste et al., 2014; Sampson and Mazmanian, 2015). A series of pro-inflammatory neurotoxins produced by intestinal microflora cross the BBB and impair the dynamic balance function of neurons in the CNS (Cryan et al., 2019). SCFA can also increase intestinal peristalsis by regulating the activity of ENS (Soret et al., 2010). Therefore, changes in SCFA concentration may lead to gastrointestinal motility disorders in patients with PD.

AD is considered a systemic disease because it is associated with neuroinflammation in the brain as well as peripheral inflammatory responses. Moreover, inflammation in the brain, which occurs many years before the appearance of plaques, is associated with Aβ production and antimicrobial responses (Bronzuoli et al., 2016; Le Page et al., 2018). Neuroinflammation is an inflammatory response of the CNS to injury or infection, accompanied by the aggregation of glial cells. During this process, chemokines, complements and pattern recognition receptors (PRRs) activate microglia and astrocytes to produce pro-inflammatory cytokines, especially IL-1β, IL-6, TNF-α (Morales et al., 2014; Calsolaro and Edison, 2016). The activated microglia in turn activate other microglia and astrocytes. An acute inflammatory response caused by activated glial cells leads to repair of the damaged areas of the brain, while chronic inflammation is usually low-grade and persistent which causes tissue degeneration. Besides, in the aging brain, chronic inflammation impairs its function of clearing abnormal proteins, leading to amyloid precursor protein (APP) accumulation, Double Helix Filament formation, and synaptic dysfunction. All of these events lead to neurodegeneration and cognitive decline (Morales et al., 2014; Lim et al., 2015). In addition, the gut flora can produce a range of metabolites, such as γ-aminobutyric acid (GABA), 5-hydroxytryptamine (5-HT), histamine and dopamine, all of which act as neurotransmitters or neurotransmitter precursors to participate in a range of mood, behavior and cognitive functions (Clarke et al., 2014; Dinan and Cryan, 2017). Previous studies have also shown that hypercholesterolemia is an early risk factor for the pathological development of amyloidosis. There are more lipid granules (or fat inclusions) in glial cells in the brain of AD patients, suggesting abnormal lipid metabolism. Population-based longitudinal studies have shown the link between high cholesterol levels and AD (Kivipelto et al., 2001).

**Figure 5:**
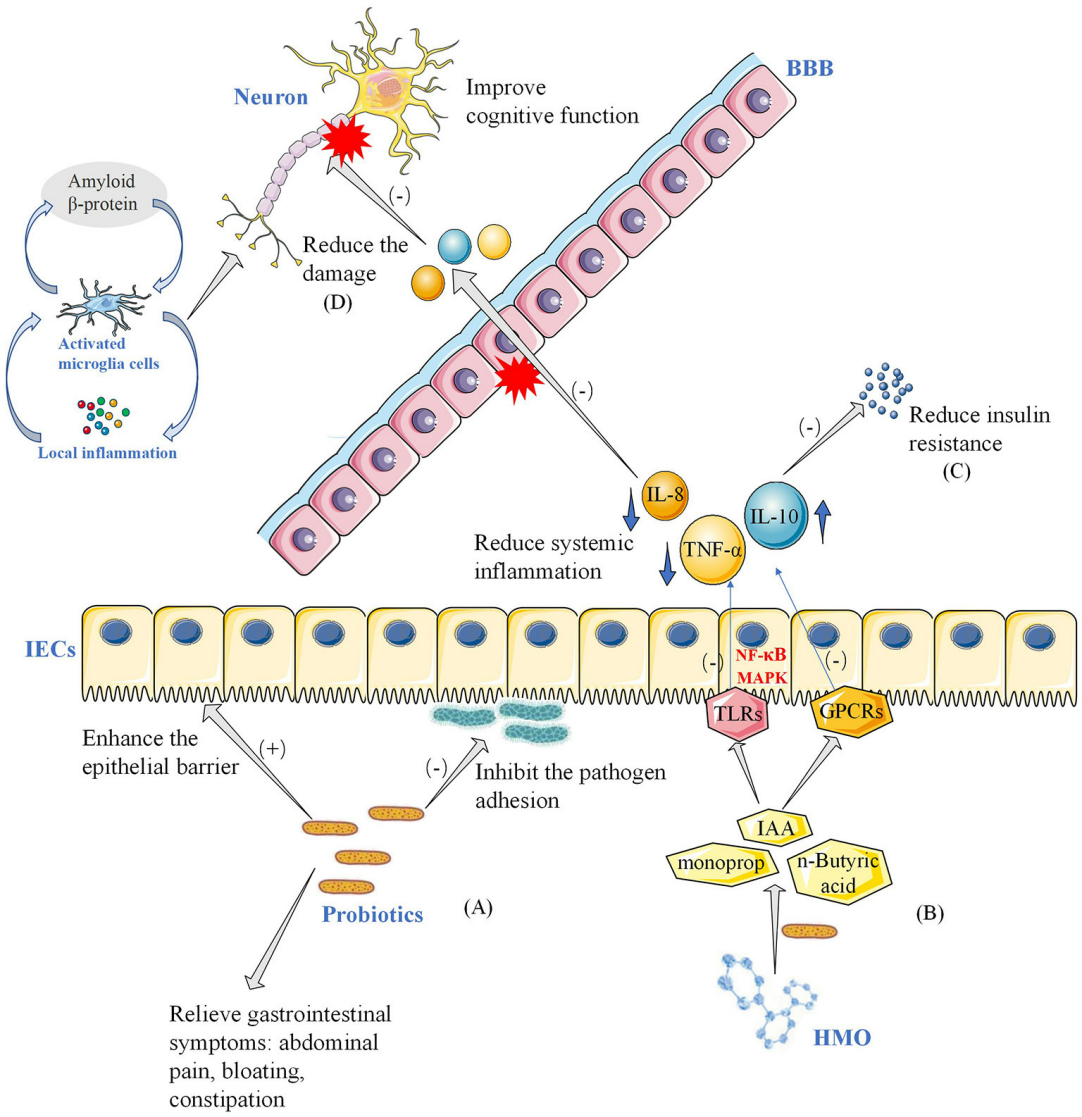
Potential mechanisms of probiotics improving neurocognitive function and gastrointestinal symptoms. **(A)** In this model, probiotics in the intestinal tract strengthen the intestinal barrier and inhibit the attachment of pathogenic bacteria to the intestinal wall, thereby improving gastrointestinal symptoms such as abdominal pain, bloating, and constipation in patients with Parkinson's disease. **(B)** Probiotics take HMO as substrate and decompose it into metabolites such as acetic acid. SCFA and other bacterial metabolites act on APC and IECs through toll-like receptors and GPCRs. Reduced systemic inflammation, including pro-inflammatory cytokines and anti-inflammatory cytokines, through NF- B and MAPK pathways. **(C)** Reduction of inflammation reduces insulin resistance on the one hand, and on the other hand **(D)** reduction of damage to the blood-brain barrier, reducing damage to neurons, and thus improving neurocognitive function. **(E)** HMO, human milk oligosaccharides; SCFA, short-chain fatty acids; GPCRs, G Protein-Coupled Receptors; APC, antigen-presenting cells; IECs, intestinal epithelial cells; NF-κB, nuclear factor kappa-light-chain-enhancer of activated B cells; MAPK, Mitogen-activated protein kinase; IL-10, interleukin 10; IL-8, interleukin 8; TNF-α, tumor necrosis factor α; BBB, blood-brain barrier; IAA, auxin; TLRs, Toll-Like Receptors; BBB, blood–brain barrier.

The World Health Organization defines probiotics as “live microorganisms, which when administered in adequate amounts, confer a health benefit on the host” (Hill et al., 2014). Although probiotic mechanisms of action are still being studied, several hypotheses have been proposed. Probiotics produce antimicrobial agents or metabolic compounds that suppress the growth of other microorganisms (Hemarajata and Versalovic, 2013). The metabolic activity of probiotics increases the amount of metabolites in the gut, such as SCFAs (Zartl et al., 2018), which represent the end products of bacterial activity in the gastrointestinal tract. These probiotics block the synthesis of hepatobiliary sterols, resulting in a decrease in the amount of lipids in the blood (Taylor and Williams, 1998; Byrne et al., 2015). They also regulate the transcription of genes involved in tight junction, improve intestinal epithelial barrier function (Anderson et al., 2010), reduce blood sugar levels and improve insulin resistance (Tabuchi et al., 2003; Bagarolli et al., 2017). The underlying mechanisms by which probiotics improve gastrointestinal function and reduce inflammatory response are shown in Figure 5. A growing body of evidence indicates that probiotics can help intestinal microflora proliferate, promote their composition shift toward a more balanced structure, and help fight pathogens by regulating the immune system (Pandey et al., 2015). The anti-inflammatory effects of probiotics in mouse models were preliminarily confirmed. Caroline Xie et al. showed that probiotics ameliorated hippocampal-dependent cognitive deficits in preclinical PD models (Xie and Prasad, 2020). Perez Visnuk et al. showed that decreased levels of inflammatory cytokines IL-6 and TNF-α in serum and increased levels of anti-inflammatory cytokines IL-10 in serum and brain tissue in the probiotic-treated PD mice (Perez Visñuk et al., 2020). A study by Athari Nik Azm et al. showed that probiotics reduced oxidative stress biomarkers and amyloid plaque formation in mice (Athari Nik Azm et al., 2018). Kobayashi et al. also showed that B. Breve A1 inhibited the expression of amyloid-β-induced hippocampal inflammation and immune response genes (Kobayashi et al., 2017). These results suggested that probiotics might play a role in health through anti-inflammatory and anti-oxidative stress. Our results showed that probiotics reduced the levels of inflammatory and oxidative stress markers (hs-CRP, MDA). In a randomized controlled trial, probiotics reduced the expression of pro-inflammatory factors (IL-1, TNF-α) genes, increased the expression of anti-inflammatory factors (TGF-β, PPAR-γ) genes, and reduced the expression of inflammatory and oxidative markers in PD patients (Borzabadi et al., 2018). It is worth noting that inflammatory and oxidative stress pathways are not unique to AD and PD, and they may play an important role in a variety of diseases, especially age-related diseases (Buford, 2017). After taking probiotics, the number of total bowel movements (CBM), the number of spontaneous bowel movements (SBM), and the Bristol Stool Scale in PD patients improved. VLDL, triglyceride and HOMA-IR were significantly improved.

Three studies about MCI showed that probiotics had no adverse effects on participants' hematological blood parameters, vital signs, and biological blood parameters, and no adverse reactions related to probiotics use were reported in the 11 included studies. Therefore, the Bifidobacterium and Lactobacillus strains used in involved studies were safe and well-tolerated.

In our study, three studies involving patients with AD used multiple strains (Akbari et al., 2016; Agahi et al., 2018; Tamtaji et al., 2019a), and the other three literatures with MCI applied a sole strain of probiotics (Hwang et al., 2019; Kobayashi et al., 2019; Xiao et al., 2020). There were significant differences between the experimental group and the control group in the cognitive function indicators MMSE and RBANS, but we could not confirm whether the single strain was effective for AD patients. In the study of Aghahi et al., AD patients were insensitive to a mixture of six strains (Agahi et al., 2018). It is suggested that the efficacy of probiotics may be related to the severity of the patient's disease, the time of intervention, the dose and the proportion of strains. Although the results showed that MMSE was statistically different between the probiotic group and placebo group, larger clinical data and longer follow-up are needed to validate its clinical value. The definition of probiotics requires an appropriate amount to obtain a health benefit, but what does is called an appropriate amount is not stated. Due to the limited information provided by the included studies, we were also unable to determine whether there was a dose-response relationship with the improvement of symptoms or determine the most appropriate dose of probiotics. Most of the included studies used a probiotic dose of 10^9^ to 10^10^ CFU as a reference. More reliable evidence is needed in the future, especially beyond conventional doses (10^9^ to 10^10^ CFU). Compared to the placebo groups, the probiotic groups all showed greater improvements in cognitive function in 12 weeks. Therefore, the present studies showed that a probiotic intervention of 12 weeks may improve cognitive function. But whether the effect can be achieved in a shorter time, or sustained over a longer period, remains to be verified.

There are also several limitations: (1) The strains, dosage and intervention time of probiotics are not the same, which will have a certain influence on the outcome. (2) The criteria for recruiting MCI subjects were different: (Kobayashi et al., 2019) used RBANS <41 as the diagnostic criteria for MCI patients; (Hwang et al., 2019) diagnosed MCI according to Diagnostic and The Statistical Manual of Mental Disorders, 5th Edition (DSM-5); (Xiao et al., 2020) took MMSE≥22 as the diagnostic criterion. (3) Among the 3 AD studies, two of them (Akbari et al., 2016; Tamtaji et al., 2019a) used MMSE as the evaluation criterion of cognition, and one (Agahi et al., 2018) evaluated cognition according to TYM, which may affect the results of the meta-analysis.

The included RCTs have some limitations. Therefore, larger RCTs with longer follow-up will be needed in the future to offer more reliable evidence. For the future RCTs, we have some suggestions as follows. Firstly, additional subgroups should be considered, including different probiotic doses, different intervention times and different severity of diseases, etc. Secondly, more indicators should be included, such as impaired cognitive related inflammatory biomarkers, S100A12, and neopterin. In addition, other cognitive assessments that may more sensitivity assess the impact of cognition rather than the global screening metric of MMSE should be conducted. At present, there are 3 ongoing RCTs on AD and 5 ongoing RCTs on PD, which will provide more evidence for future studies. The information of these RCTs is shown in the Supplementary Material.

## Conclusion

This meta-analysis suggested that probiotics could enhance cognitive function in patients with AD and MCI and improve gastrointestinal symptoms in patients with PD. Probiotics may be involved in reducing biomarkers of inflammation and oxidative stress. However, the current RCTs still have some limitations, and larger RCTs with longer follow-up will be needed in the future to provide more reliable evidence.

## Data Availability Statement

The original contributions presented in the study are included in the article/Supplementary Material, further inquiries can be directed to the corresponding author/s.

## Author Contributions

SX and C-CT: conceptualization and design of the study, collection and analysis of the data, drafting and revision of the manuscript, and prepared all the figures. J-LJ and SL: collection and analysis of the data, and revision of the manuscript. WX, X-PC, and LT: revision of the manuscript. All authors contributed to the article and approved the submitted version.

## Funding

This work was supported by grants from the National Natural Science Foundation of China (81901121 and 82001136), and the Qilu Health Care Outstanding Young Talent Program.

## Conflict of Interest

The authors declare that the research was conducted in the absence of any commercial or financial relationships that could be construed as a potential conflict of interest.

## Publisher's Note

All claims expressed in this article are solely those of the authors and do not necessarily represent those of their affiliated organizations, or those of the publisher, the editors and the reviewers. Any product that may be evaluated in this article, or claim that may be made by its manufacturer, is not guaranteed or endorsed by the publisher.

## References

[B1] Agahi A. Hamidi G. A. Daneshvar R. Hamdieh M. Soheili M. Alinaghipour A. . (2018). Does severity of Alzheimer's disease contribute to its responsiveness to modifying gut microbiota? A double blind clinical trial. Front. Neurol. 9:662. 10.3389/fneur.2018.00662 30158897 PMC6104449

[B2] Akbari E. Asemi Z. Daneshvar Kakhaki R. Bahmani F. Kouchaki E. Tamtaji O. R. . (2016). Effect of probiotic supplementation on cognitive function and metabolic status in Alzheimer's disease: a randomized, double-blind and controlled trial. Front. Aging Neurosci. 8:256. 10.3389/fnagi.2016.00256 27891089 PMC5105117

[B3] Alkasir R. Li J. Li X. Jin M. Zhu B. (2017). Human gut microbiota: the links with dementia development. Protein Cell 8, 90–102. 10.1007/s13238-016-0338-6 27866330 PMC5291774

[B4] Anderson R. C. Cookson A. L. McNabb W. C. Park Z. McCann M. J. Kelly W. J. . (2010). Lactobacillus plantarum MB452 enhances the function of the intestinal barrier by increasing the expression levels of genes involved in tight junction formation. BMC Microbiol. 10:316. 10.1186/1471-2180-10-316 21143932 PMC3004893

[B5] Athari Nik Azm S. Djazayeri A. Safa M. Azami K. Ahmadvand B. Sabbaghziarani F. . (2018). Lactobacilli and bifidobacteria ameliorate memory and learning deficits and oxidative stress in β-amyloid (1-42) injected rats. Appl. Physiol. Nutr. Metab. 43, 718–26. 10.1139/apnm-2017-0648 29462572

[B6] Bagarolli R. A. Tobar N. Oliveira A. G. Araújo T. G. Carvalho B. M. Rocha G. Z. . (2017). Probiotics modulate gut microbiota and improve insulin sensitivity in DIO mice. J. Nutr. Biochem. 50, 16–25. 10.1016/j.jnutbio.2017.08.006 28968517

[B7] Bangen K. J. Clark A. L. Edmonds E. C. Evangelista N. D. Werhane M. L. Thomas K. R. . (2017). Cerebral blood flow and Amyloid-β interact to affect memory performance in cognitively normal older adults. Front. Aging Neurosci. 9:181. 10.3389/fnagi.2017.00181 28642699 PMC5463038

[B8] Barichella M. Pacchetti C. Bolliri C. Cassani E. Iorio L. Pusani C. . (2016). Probiotics and prebiotic fiber for constipation associated with Parkinson disease: an RCT. Neurology 87, 1274–1280. 10.1212/WNL.0000000000003127 27543643

[B9] Borzabadi S. Oryan S. Eidi A. Aghadavod E. Daneshvar Kakhaki R. Tamtaji O. R. . (2018). The effects of probiotic supplementation on gene expression related to inflammation, insulin and lipid in patients with Parkinson's disease: a randomized, double-blind, placebocontrolled trial. Arch. Iran Med. 21, 289–295. 30041526

[B10] Braniste V. Al-Asmakh M. Kowal C. Anuar F. Abbaspour A. Tóth M. . (2014). The gut microbiota influences blood-brain barrier permeability in mice. Sci. Transl. Med. 6:263ra158. 10.1126/scitranslmed.3009759 25411471 PMC4396848

[B11] Bronzuoli M. R. Iacomino A. Steardo L. Scuderi C. (2016). Targeting neuroinflammation in Alzheimer's disease. J. Inflamm. Res. 9, 199–208. 10.2147/JIR.S86958 27843334 PMC5098782

[B12] Buford T. W.. (2017). (Dis)Trust your gut: the gut microbiome in age-related inflammation, health, and disease. Microbiome 5:80. 10.1186/s40168-017-0296-0 28709450 PMC5512975

[B13] Byrne C. S. Chambers E. S. Morrison D. J. Frost G. (2015). The role of short chain fatty acids in appetite regulation and energy homeostasis. Int. J. Obes. 39, 1331–1338. 10.1038/ijo.2015.84 25971927 PMC4564526

[B14] Calsolaro V. Edison P. (2016). Neuroinflammation in Alzheimer's disease: current evidence and future directions. Alzheimers Dement. 12, 719–732. 10.1016/j.jalz.2016.02.010 27179961

[B15] Caracciolo B. Xu W. Collins S. Fratiglioni L. (2014). Cognitive decline, dietary factors and gut-brain interactions. Mech. Ageing Dev. 136–137, 59–69. 10.1016/j.mad.2013.11.011 24333791

[B16] Chunchai T. Thunapong W. Yasom S. Wanchai K. Eaimworawuthikul S. Metzler G. . (2018). Decreased microglial activation through gut-brain axis by prebiotics, probiotics, or synbiotics effectively restored cognitive function in obese-insulin resistant rats. J. Neuroinflammation 15:11. 10.1186/s12974-018-1055-2 29316965 PMC5761137

[B17] Cirstea M. S. Yu A. C. Golz E. Sundvick K. Kliger D. Radisavljevic N. . (2020). Microbiota composition and metabolism are associated with gut function in Parkinson's disease. Mov. Disord. 35, 1208–1217. 10.1002/mds.28052 32357258

[B18] Clarke G. Stilling R. M. Kennedy P. J. Stanton C. Cryan J. F. Dinan T. G. (2014). Minireview: gut microbiota: the neglected endocrine organ. Mol. Endocrinol. 28, 1221–1238. 10.1210/me.2014-1108 24892638 PMC5414803

[B19] Cryan J. F. O'Riordan K. J. Cowan C. S. M. Sandhu K. V. Bastiaanssen T. F. S. Boehme M. . (2019). The microbiota-gut-brain axis. Physiol. Rev. 99, 1877–2013. 10.1152/physrev.00018.2018 31460832

[B20] Cummings J. Salloway S. (2021). Aducanumab: appropriate use recommendations. Alzheimers Dement. 8, 398–410. 10.1002/alz.12444 34585212 PMC8835345

[B21] Cumpston M. Li T. Page M. J. Chandler J. Welch V. A. Higgins J. P. . (2019). Updated guidance for trusted systematic reviews: a new edition of the Cochrane Handbook for Systematic Reviews of Interventions. Cochrane Database Syst. Rev. 10:Ed000142. 10.1002/14651858.ED000142 31643080 PMC10284251

[B22] Dinan T. G. Cryan J. F. (2017). Gut instincts: microbiota as a key regulator of brain development, ageing and neurodegeneration. J. Physiol. 595, 489–503. 10.1113/JP273106 27641441 PMC5233671

[B23] Dugger B. N. Dickson D. W. (2017). Pathology of neurodegenerative diseases. Cold Spring Harb. Perspect. Biol. 9:a028035. 10.1101/cshperspect.a028035 28062563 PMC5495060

[B24] Franceschi C. Campisi J. (2014). Chronic inflammation (inflammaging) and its potential contribution to age-associated diseases. J. Gerontol. A Biol. Sci. Med. Sci. 69(Suppl. 1), S4–9. 10.1093/gerona/glu057 24833586

[B25] Frasca D. Blomberg B. B. (2016). Inflammaging decreases adaptive and innate immune responses in mice and humans. Biogerontology 17, 7–19. 10.1007/s10522-015-9578-8 25921609 PMC4626429

[B26] Fratiglioni L. Qiu C. (2009). Prevention of common neurodegenerative disorders in the elderly. Exp. Gerontol. 44, 46–50. 10.1016/j.exger.2008.06.006 18620039

[B27] Georgescu D. Ancusa O. E. Georgescu L. A. Ionita I. Reisz D. (2016). Nonmotor gastrointestinal disorders in older patients with Parkinson's disease: is there hope? Clin. Interv. Aging 11, 1601–1608. 10.2147/CIA.S106284 27956826 PMC5113937

[B28] Guarner F. Malagelada J. R. (2003). Gut flora in health and disease. Lancet 361, 512–519. 10.1016/S0140-6736(03)12489-0 12583961

[B29] Heemels M. T.. (2016). Neurodegenerative diseases. Nature 539:179. 10.1038/539179a 27830810

[B30] Hemarajata P. Versalovic J. (2013). Effects of probiotics on gut microbiota: mechanisms of intestinal immunomodulation and neuromodulation. Therap. Adv. Gastroenterol. 6, 39–51. 10.1177/1756283X12459294 23320049 PMC3539293

[B31] Hill C. Guarner F. Reid G. Gibson G. R. Merenstein D. J. Pot B. . (2014). Expert consensus document. The International Scientific Association for Probiotics and Prebiotics consensus statement on the scope and appropriate use of the term probiotic. Nat. Rev. Gastroenterol. Hepatol. 11, 506–514. 10.1038/nrgastro.2014.66 24912386

[B32] Hwang Y. H. Park S. Paik J. W. Chae S. W. Kim D. H. Jeong D. G. . (2019). Efficacy and safety of lactobacillus plantarum C29-Fermented Soybean (DW2009) in individuals with mild cognitive impairment: a 12-week, multi-center, randomized, double-blind, placebo-controlled clinical trial. Nutrients 11:305. 10.3390/nu11020305 30717153 PMC6412773

[B33] Jucker M. Walker L. C. (2018). Propagation and spread of pathogenic protein assemblies in neurodegenerative diseases. Nat. Neurosci. 21, 1341–1349. 10.1038/s41593-018-0238-6 30258241 PMC6375686

[B34] Kesika P. Suganthy N. Sivamaruthi B. S. Chaiyasut C. (2021). Role of gut-brain axis, gut microbial composition, and probiotic intervention in Alzheimer's disease. Life Sci. 264:118627. 10.1016/j.lfs.2020.118627 33169684

[B35] Kivipelto M. Helkala E. L. Laakso M. P. Hänninen T. Hallikainen M. Alhainen K. . (2001). Midlife vascular risk factors and Alzheimer's disease in later life: longitudinal, population based study. BMJ 322, 1447–1451. 10.1136/bmj.322.7300.1447 11408299 PMC32306

[B36] Kobayashi Y. Kuhara T. Oki M. Xiao J. Z. (2019). Effects of *Bifidobacterium breve* A1 on the cognitive function of older adults with memory complaints: a randomised, double-blind, placebo-controlled trial. Benef. Microbes. 10, 511–520. 10.3920/BM2018.0170 31090457

[B37] Kobayashi Y. Sugahara H. Shimada K. Mitsuyama E. Kuhara T. Yasuoka A. . (2017). Therapeutic potential of *Bifidobacterium breve* strain A1 for preventing cognitive impairment in Alzheimer's disease. Sci. Rep. 7:13510. 10.1038/s41598-017-13368-2 29044140 PMC5647431

[B38] Lambert J. C. Heath S. Even G. Campion D. Sleegers K. Hiltunen M. . (2009). Genome-wide association study identifies variants at CLU and CR1 associated with Alzheimer's disease. Nat. Genet. 41, 1094–1099. 10.1038/ng.439 19734903

[B39] Le Page A. Dupuis G. Frost E. H. Larbi A. Pawelec G. Witkowski J. M. . (2018). Role of the peripheral innate immune system in the development of Alzheimer's disease. Exp. Gerontol. 107:59–66. 10.1016/j.exger.2017.12.019 29275160

[B40] Li B. He Y. Ma J. Huang P. Du J. Cao L. . (2019). Mild cognitive impairment has similar alterations as Alzheimer's disease in gut microbiota. Alzheimers Dement. 15, 1357–1366. 10.1016/j.jalz.2019.07.002 31434623

[B41] Li W. Wu X. Hu X. Wang T. Liang S. Duan Y. . (2017). Structural changes of gut microbiota in Parkinson's disease and its correlation with clinical features. Sci. China Life Sci. 60, 1223–1233. 10.1007/s11427-016-9001-4 28536926

[B42] Lim S. L. Rodriguez-Ortiz C. J. Kitazawa M. (2015). Infection, systemic inflammation, and Alzheimer's disease. Microbes. Infect. 17, 549–556. 10.1016/j.micinf.2015.04.004 25912134

[B43] Lynch S. V. Pedersen O. (2016). The human intestinal microbiome in health and disease. N. Engl. J. Med. 375, 2369–2379. 10.1056/NEJMra1600266 27974040

[B44] Mitsuma T. Odajima H. Momiyama Z. Watanabe K. Masuguchi M. Sekine T. . (2008). Enhancement of gene expression by a peptide p(CHWPR) produced by *Bifidobacterium lactis* BB-12. Microbiol. Immunol. 52, 144–155. 10.1111/j.1348-0421.2008.00022.x 18402596

[B45] Morales I. Guzmán-Martínez L. Cerda-Troncoso C. Farías G. A. Maccioni R. B. (2014). Neuroinflammation in the pathogenesis of Alzheimer's disease. a rational framework for the search of novel therapeutic approaches. Front. Cell Neurosci. 8:112. 10.3389/fncel.2014.00112 24795567 PMC4001039

[B46] Morris M. C. Tangney C. C. Wang Y. Sacks F. M. Barnes L. L. Bennett D. A. . (2015). MIND diet slows cognitive decline with aging. Alzheimers Dement. 11, 1015–1022. 10.1016/j.jalz.2015.04.011 26086182 PMC4581900

[B47] Noseworthy M. D. Bray T. M. (1998). Effect of oxidative stress on brain damage detected by MRI and *in vivo* 31P-NMR. Free Radic. Biol. Med. 24, 942–951. 10.1016/S0891-5849(97)00383-3 9607604

[B48] Pandey K. R. Naik S. R. Vakil B. V. (2015). Probiotics, prebiotics and synbiotics- a review. J. Food Sci. Technol. 52, 7577–7587. 10.1007/s13197-015-1921-1 26604335 PMC4648921

[B49] Perez Visñuk D. Savoy de Giori G. LeBlanc J. G. de Moreno de LeBlanc A. (2020). Neuroprotective effects associated with immune modulation by selected lactic acid bacteria in a Parkinson's disease model. Nutrition 79–80:110995. 10.1016/j.nut.2020.110995 32977125

[B50] Petersen R. C.. (2004). Mild cognitive impairment as a diagnostic entity. J. Intern. Med. 256, 183–194. 10.1111/j.1365-2796.2004.01388.x 15324362

[B51] Sampson T. R. Mazmanian S. K. (2015). Control of brain development, function, and behavior by the microbiome. Cell Host Microbe. 17, 565–576. 10.1016/j.chom.2015.04.011 25974299 PMC4442490

[B52] Scheperjans F. Aho V. Pereira P. A. Koskinen K. Paulin L. Pekkonen E. . (2015). Gut microbiota are related to Parkinson's disease and clinical phenotype. Mov. Disord. 30, 350–358. 10.1002/mds.26069 25476529

[B53] Skonieczna-Zydecka K. Marlicz W. Misera A. Koulaouzidis A. Łoniewski I. (2018). Microbiome-the missing link in the gut-brain axis: focus on its role in gastrointestinal and mental health. J. Clin. Med. 7:521. 10.3390/jcm7120521 30544486 PMC6306769

[B54] Soret R. Chevalier J. De Coppet P. Poupeau G. Derkinderen P. Segain J. P. . (2010). Short-chain fatty acids regulate the enteric neurons and control gastrointestinal motility in rats. Gastroenterology 138, 1772–1782. 10.1053/j.gastro.2010.01.053 20152836

[B55] Sperling R. A. Jack C. R. Jr. Black S. E. Frosch M. P. Greenberg S. M. Hyman B. T. . (2011). Amyloid-related imaging abnormalities in amyloid-modifying therapeutic trials: recommendations from the Alzheimer's Association Research Roundtable Workgroup. Alzheimers Dement. 7, 367–385. 10.1016/j.jalz.2011.05.2351 21784348 PMC3693547

[B56] Spielman L. J. Gibson D. L. Klegeris A. (2018). Unhealthy gut, unhealthy brain: the role of the intestinal microbiota in neurodegenerative diseases. Neurochem. Int. 120, 149–163. 10.1016/j.neuint.2018.08.005 30114473

[B57] Stephenson J. Nutma E. van der Valk P. Amor S. (2018). Inflammation in CNS neurodegenerative diseases. Immunology 154, 204–219. 10.1111/imm.12922 29513402 PMC5980185

[B58] Tabuchi M. Ozaki M. Tamura A. Yamada N. Ishida T. Hosoda M. . (2003). Antidiabetic effect of Lactobacillus GG in streptozotocin-induced diabetic rats. Biosci. Biotechnol. Biochem. 67, 1421–1424. 10.1271/bbb.67.1421 12843677

[B59] Tamtaji O. R. Heidari-Soureshjani R. Mirhosseini N. Kouchaki E. Bahmani F. Aghadavod E. . (2019a). Probiotic and selenium co-supplementation, and the effects on clinical, metabolic and genetic status in Alzheimer's disease: a randomized, double-blind, controlled trial. Clin. Nutr. 38, 2569–2575. 10.1016/j.clnu.2018.11.034 30642737

[B60] Tamtaji O. R. Taghizadeh M. Daneshvar Kakhaki R. Kouchaki E. Bahmani F. Borzabadi S. . (2019b). Clinical and metabolic response to probiotic administration in people with Parkinson's disease: a randomized, double-blind, placebo-controlled trial. Clin. Nutr. 38, 1031–1035. 10.1016/j.clnu.2018.05.018 29891223

[B61] Tan A. H. Lim S. Y. Chong K. K. A Manap M. A. A. Hor J. W. Lim J. L. . (2021). Probiotics for constipation in Parkinson disease: a randomized placebo-controlled study. Neurology 96:e772–e82. 10.1212/WNL.0000000000010998 33046607

[B62] Taylor G. R. Williams C. M. (1998). Effects of probiotics and prebiotics on blood lipids. Br. J. Nutr. 80, S225–S230. 10.1017/S0007114500006073 9924289

[B63] Thursby E. Juge N. (2017). Introduction to the human gut microbiota. Biochem. J. 474, 1823–1836. 10.1042/BCJ20160510 28512250 PMC5433529

[B64] Unger M. M. Spiegel J. Dillmann K. U. Grundmann D. Philippeit H. Bürmann J. . (2016). Short chain fatty acids and gut microbiota differ between patients with Parkinson's disease and age-matched controls. Parkinsonism Relat. Disord. 32, 66–72. 10.1016/j.parkreldis.2016.08.019 27591074

[B65] Wadhwa R. Gupta R. Maurya P. K. (2018). Oxidative stress and accelerated aging in neurodegenerative and neuropsychiatric disorder. Curr. Pharm. Des. 24, 4711–4725. 10.2174/1381612825666190115121018 30644343

[B66] Wallace C. J. K. Milev R. (2017). The effects of probiotics on depressive symptoms in humans: a systematic review. Ann. Gen. Psychiatry 16:14. 10.1186/s12991-017-0138-2 28239408 PMC5319175

[B67] Wang H. Lee I. S. Braun C. Enck P. (2016). Effect of probiotics on central nervous system functions in animals and humans: a systematic review. J. Neurogastroenterol. Motil. 22, 589–605. 10.5056/jnm16018 27413138 PMC5056568

[B68] Westfall S. Lomis N. Kahouli I. Dia S. Y. Singh S. P. Prakash S. (2017). Microbiome, probiotics and neurodegenerative diseases: deciphering the gut brain axis. Cell Mol. Life Sci. 74, 3769–3787. 10.1007/s00018-017-2550-9 28643167 PMC11107790

[B69] Xiao J. Katsumata N. Bernier F. Ohno K. Yamauchi Y. Odamaki T. . (2020). Probiotic *Bifidobacterium breve* in improving cognitive functions of older adults with suspected mild cognitive impairment: a randomized, double-blind, placebo-controlled trial. J. Alzheimers Dis. 77, 139–147. 10.3233/JAD-200488 32623402 PMC7592675

[B70] Xie C. Prasad A. A. (2020). Probiotics treatment improves hippocampal dependent cognition in a rodent model of Parkinson's disease. Microorganisms 8:1661. 10.3390/microorganisms8111661 33120961 PMC7692862

[B71] Zartl B. Silberbauer K. Loeppert R. Viernstein H. Praznik W. Mueller M. (2018). Fermentation of non-digestible raffinose family oligosaccharides and galactomannans by probiotics. Food Funct. 9, 1638–1646. 10.1039/C7FO01887H 29465736

