# Supplementary material for: Efficacy and Safety of Probiotics for the Treatment of Alzheimer's Disease, Mild Cognitive Impairment, and Parkinson's Disease: A Systematic Review and Meta-Analysis
Source: Front Aging Neurosci. 2022 Feb 3;14:730036. doi: 10.3389/fnagi.2022.730036 (PMC8851038; doi:10.3389/fnagi.2022.730036)
Supplement: Supplementary file 1 [file Data_Sheet_1.PDF]

Supplementary Figure 1. Publication bias.

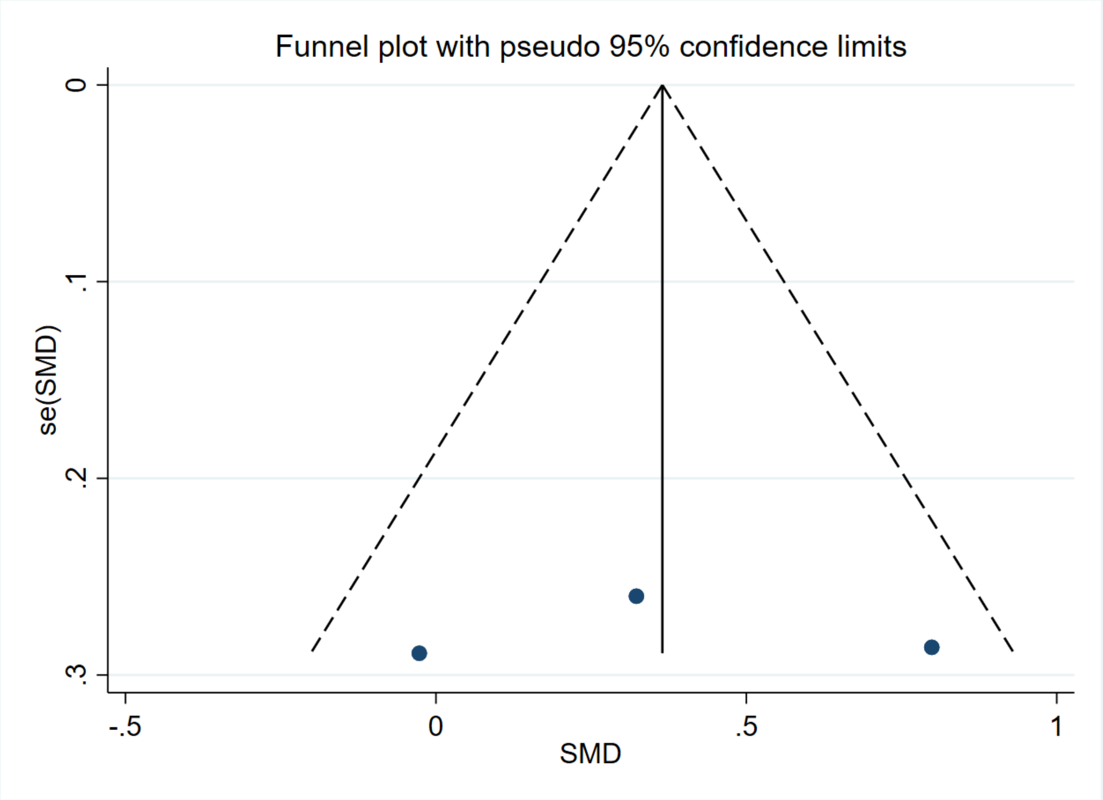

Begg's funnel plot and test

adj. Kendall's Score (P-Q) = -1

Std.dev. of Score = 1.91

Number of Studies = 3

Z = 0.52

Pr > | z | = 0.602

Z = 0.00 (Continuity Corrected)

Pr > | z | = 1.000 (continuity corrected)

Egger's test

Std_Eff	Coef.	Std. Err.	t	P>|t|	[95% Conf. Interval]
slope	2876165.	6.767952	0.04	0.973	85.70737
bias	2785545.	24.40544	0.01	0.993	309.8219

Supplementary Figure 2. Sensitivity analysis (Leave-one-out influence analysis).

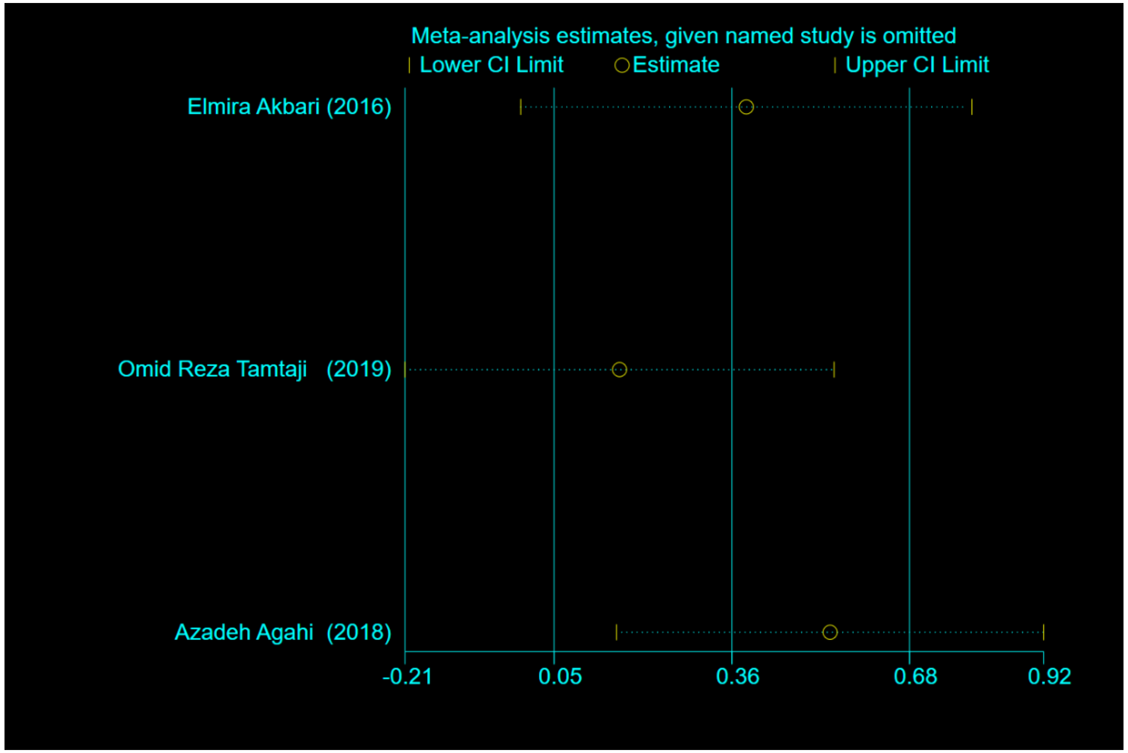

Supplementary Table 1. Summary of the ongoing Alzheimer’s disease, mild cognitive impairment and Parkinson's disease prevention RCTs

Study	Estimated enrollment	Status	Estimated Completion Date	Age	Cognitive function	Daily dose	Duration	Primary outcomes	Allocation	Masking	Intervention Model
NCT03991195	90	Recruiting	December 2021	55 Years and older	MCI	None	3 months	1.Improvement of Montreal cognitive assessment (MoCA)<br>2.Improvement of Auditory Verbal Learning Test (AVLT)<br>3.Changes of intestinal microbial flora diversity	Randomized	Double Blind	Parallel Assignment
NCT03847714	58	Recruiting	December 2021	18 Years and older	Dementia of Alzheimer type and mixed type	7.5×10^9 CFU/g	6 months	1.Butyrate producing bacteria	Randomized	Double Blind	Parallel Assignment
NCT03968133	72	Recruiting	December 2022	40 Years to 80 Years	Parkinson's disease	2.5×10^9 CFU/g	13 weeks	1. Parkinson's Anxiety Scale (PAS)	Randomized	Double Blind	Parallel Assignment

NCT04293159	30	Recruiting	December 31, 2020	18 Years to 75 Years	Parkinson Disease	None	12 weeks	1. Satisfaction with therapy<br>2. Improvement after treatment<br>3. Neuropsychological function	N/A	None (Open Label)	Single Group Assignment
NCT04722211	120	Recruiting	June 30, 2023	45 Years to 80 Years	Parkinson Disease	$>1\times10^{10}$ CFU/capsule	12 weeks	1. UPDRS III<br>2. MHY<br>3. TUG	Randomized	Double Blind	Parallel Assignment

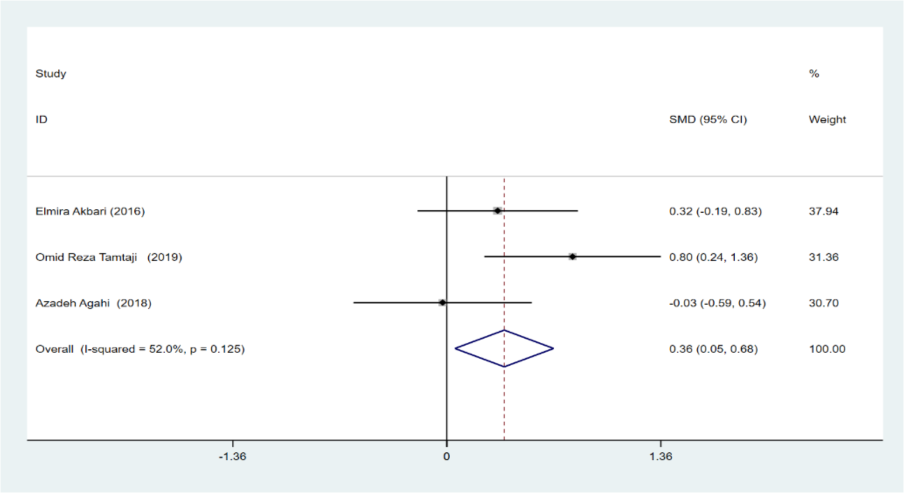

MMSE (AD)

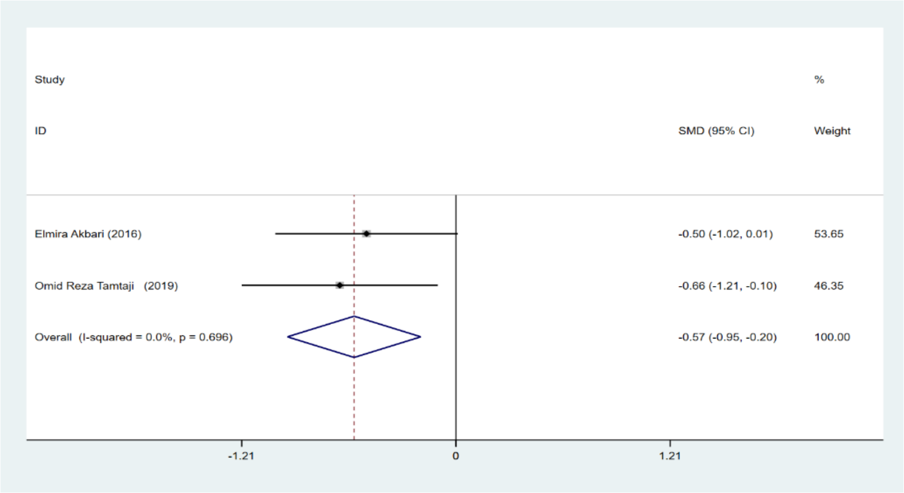

hs-CRP (AD)

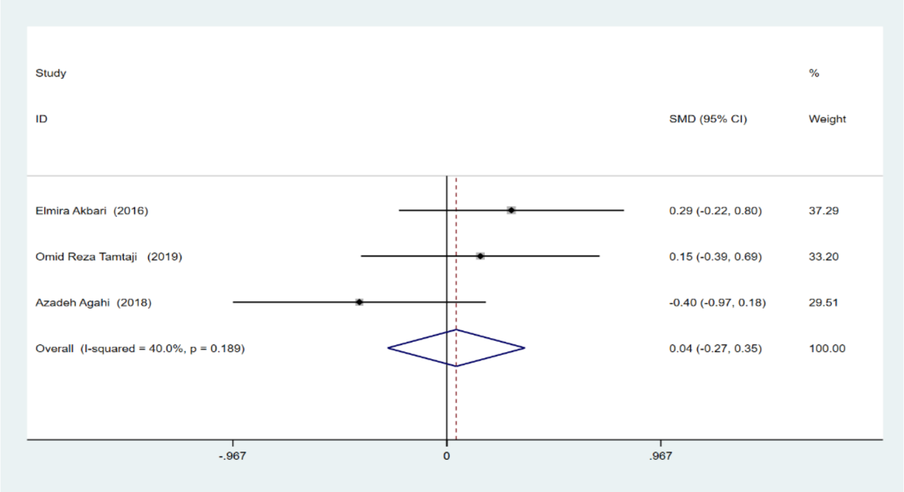

GSH (AD)

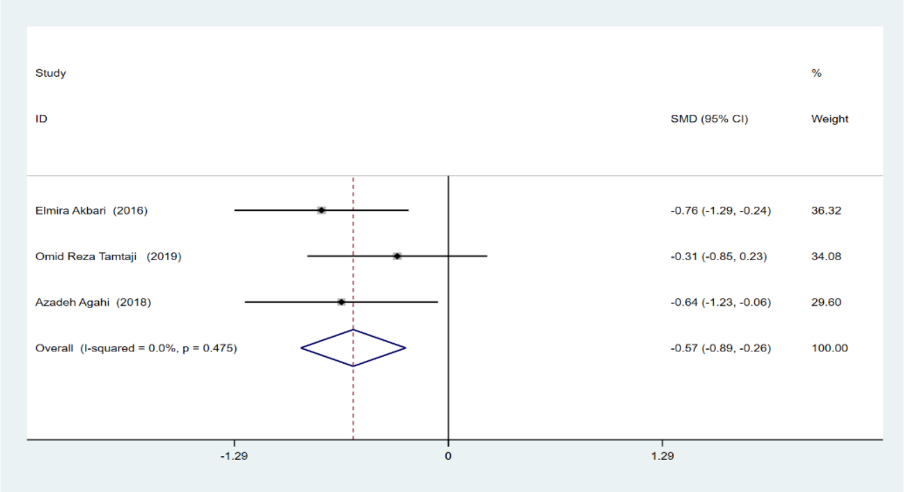

MDA (AD)

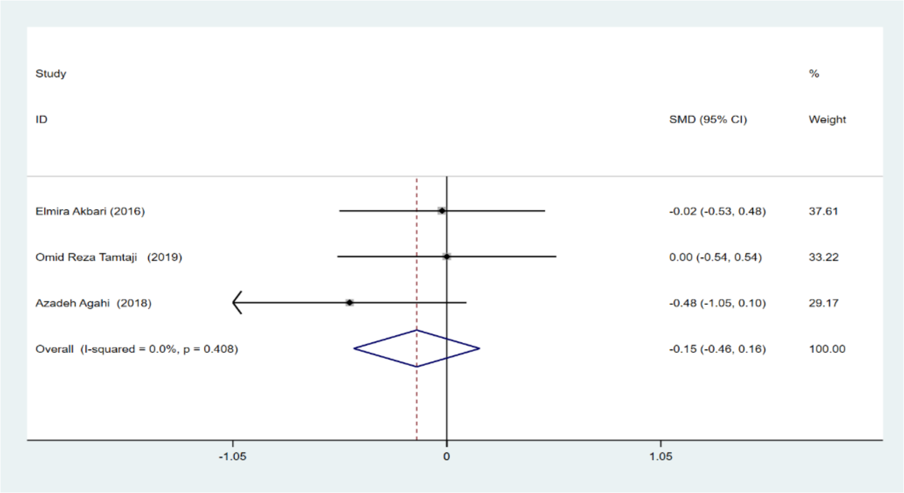

NO (AD)

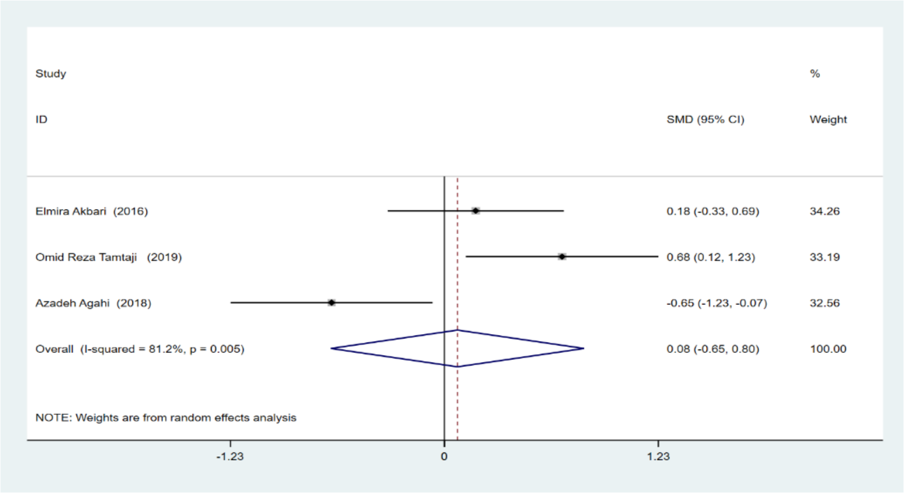

TAC (AD)

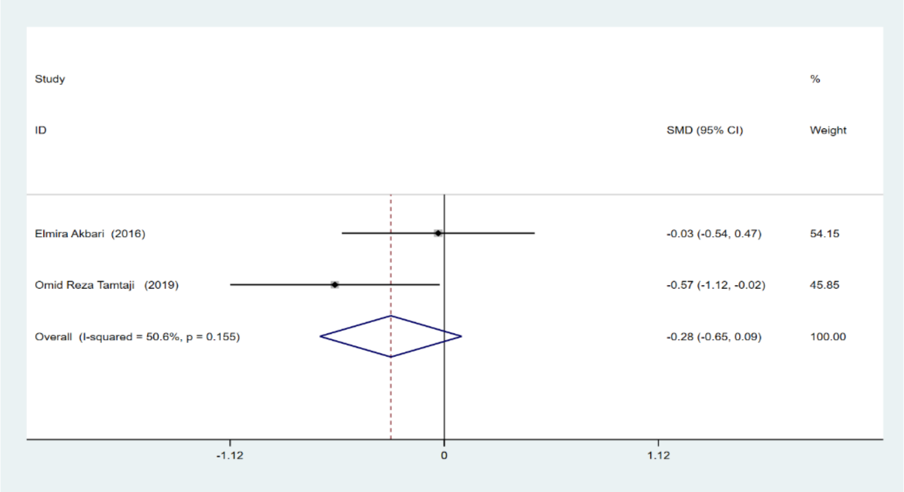

FPG (AD)

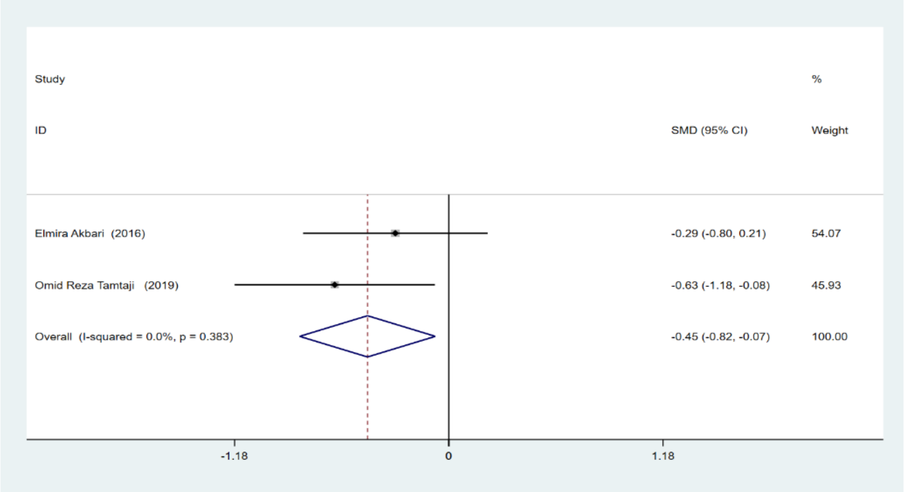

HOMA-IR (AD)

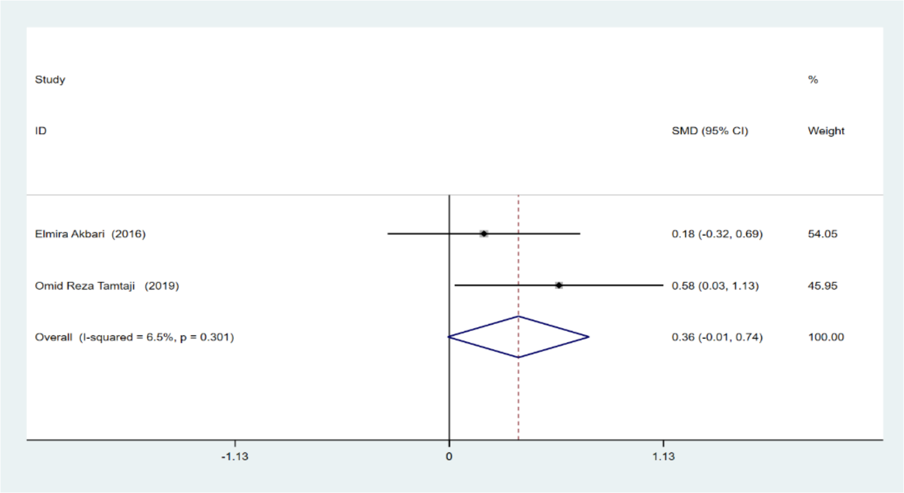

QUICKI (AD)

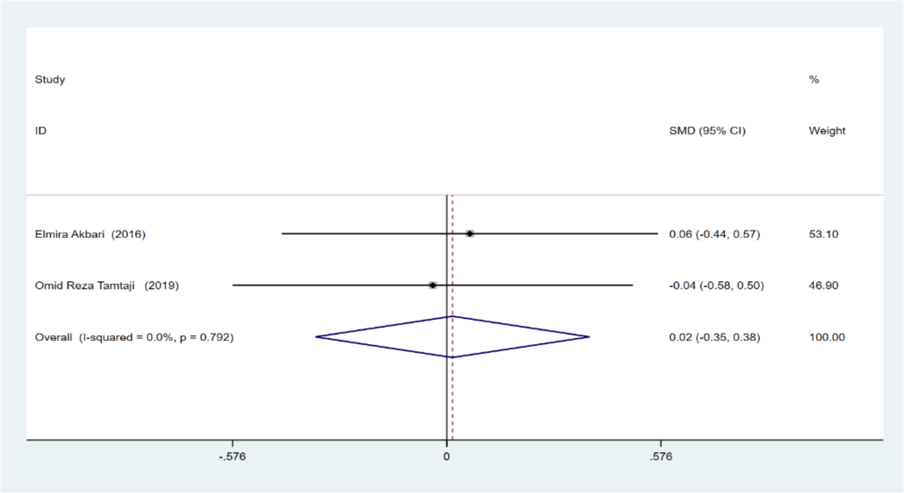

LDL (AD)

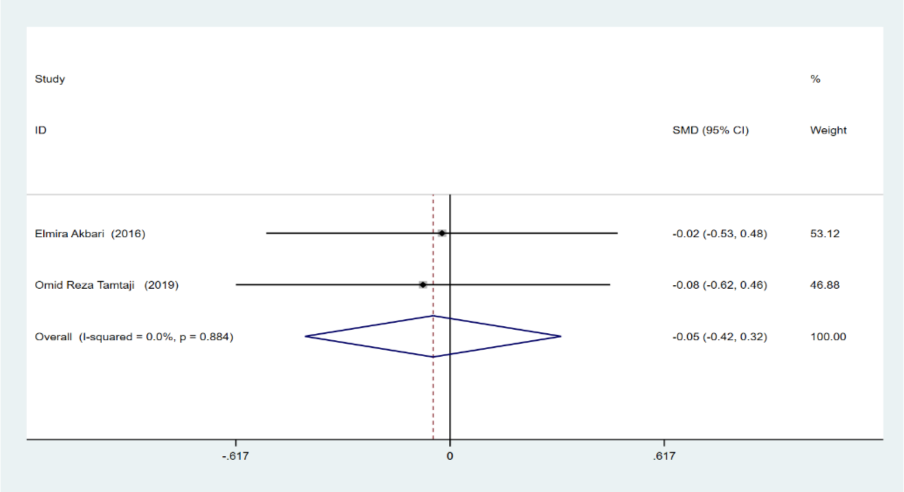

HDL (AD)

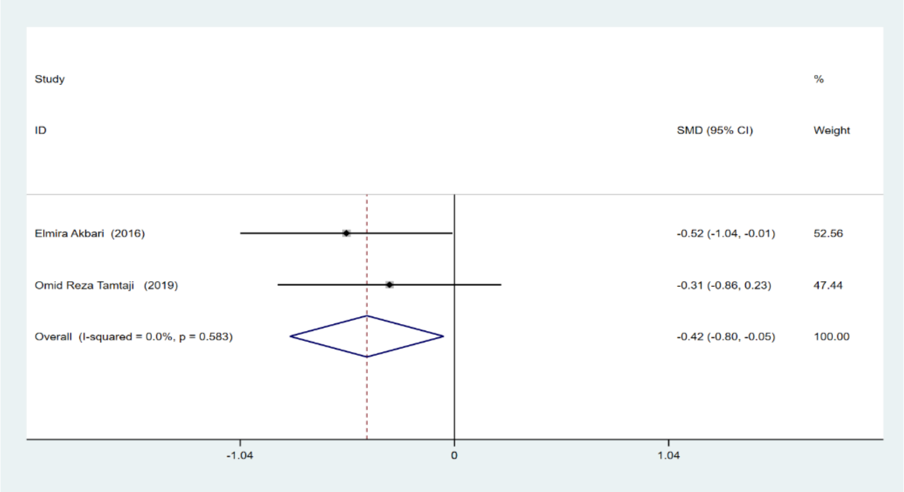

VLDL (AD)

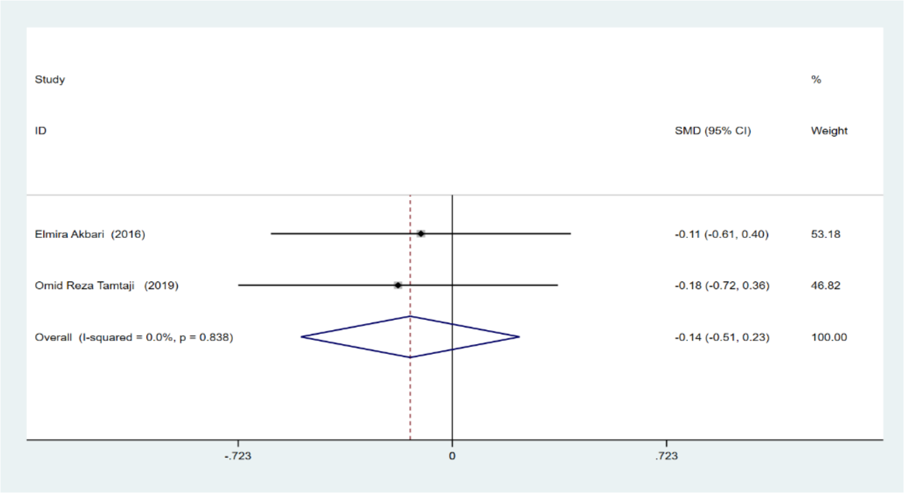

Total Cholesterol (AD)

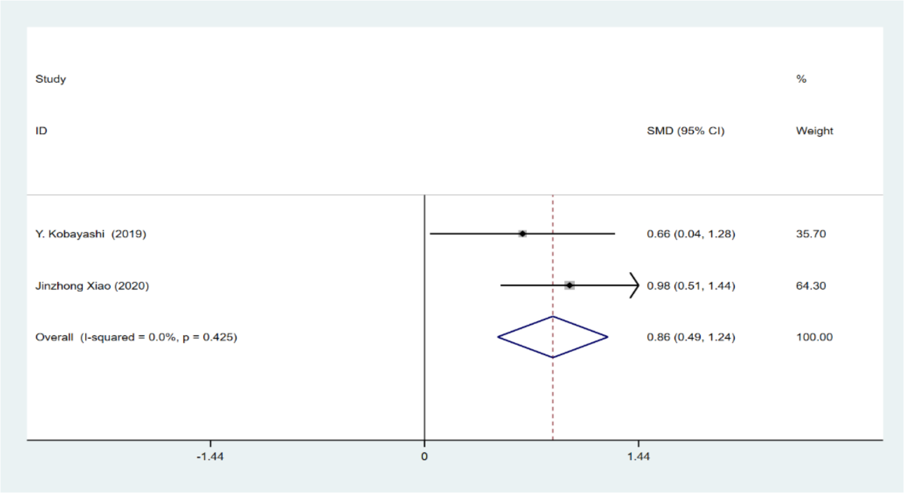

RBANS (MCI)

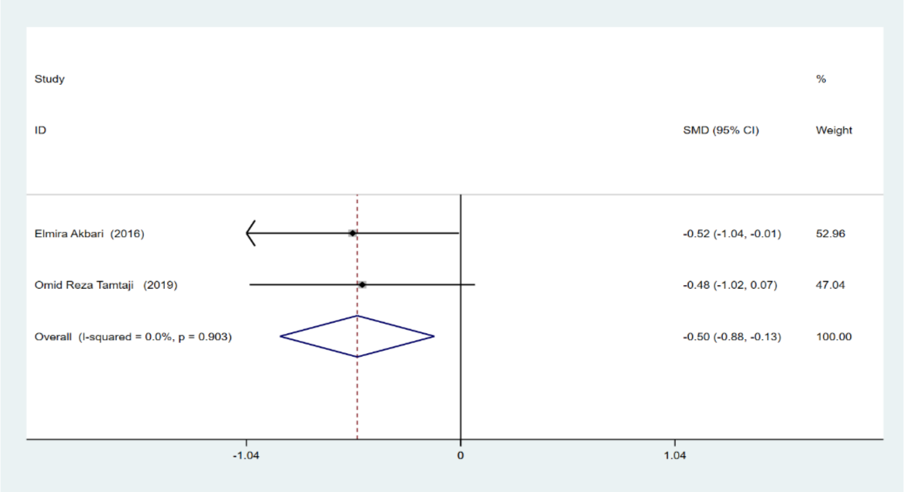

Triglycerides (AD)

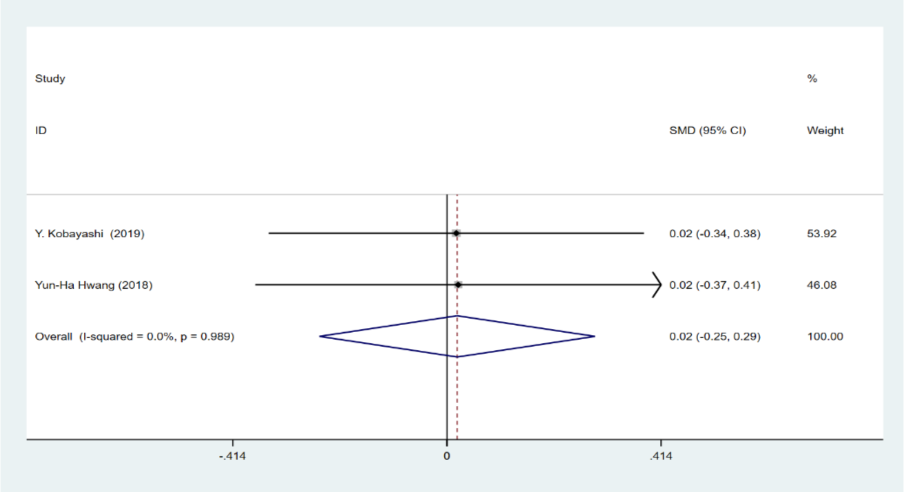

Total Cholesterol (MCI)

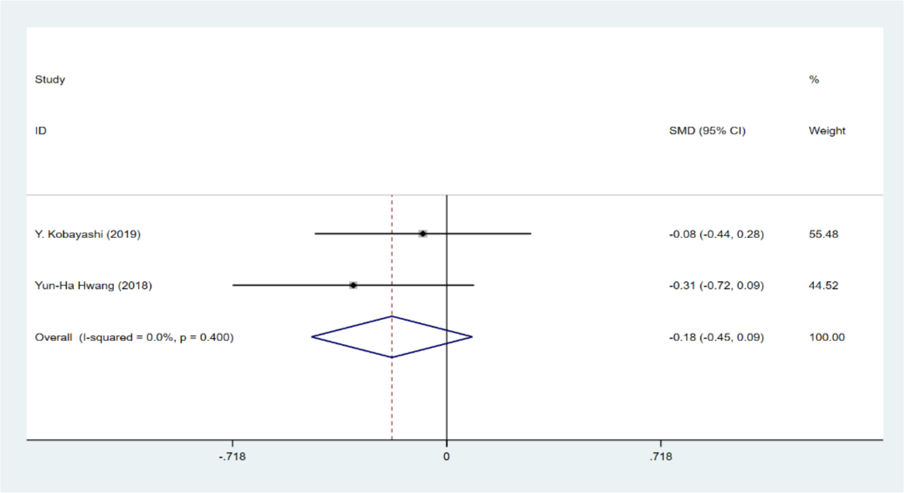

ALB (MCI)

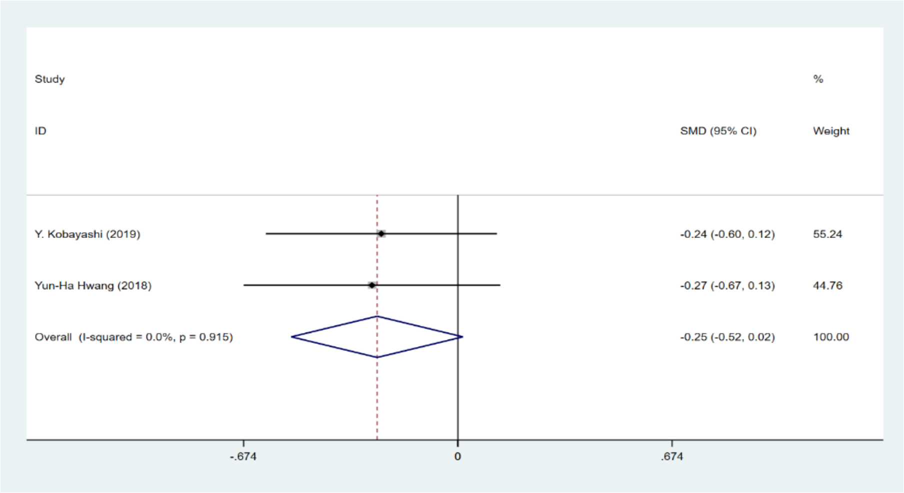

ALT (MCI)

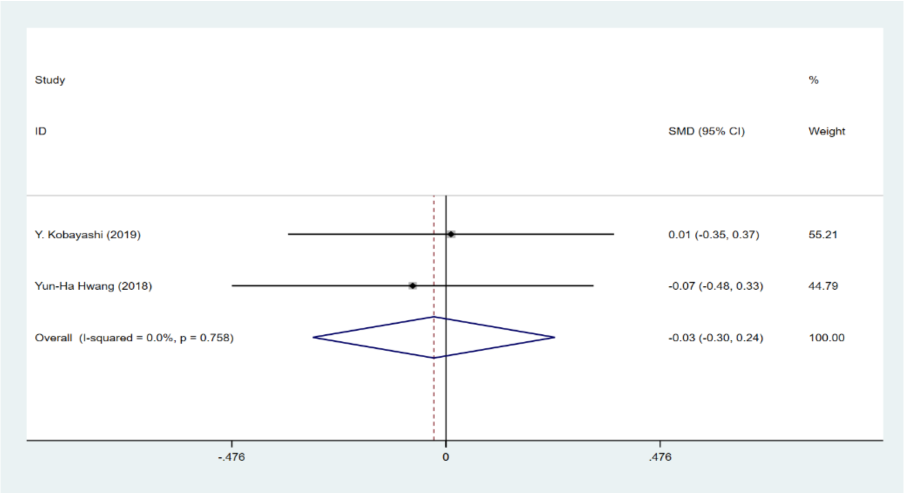

ALP (MCI)

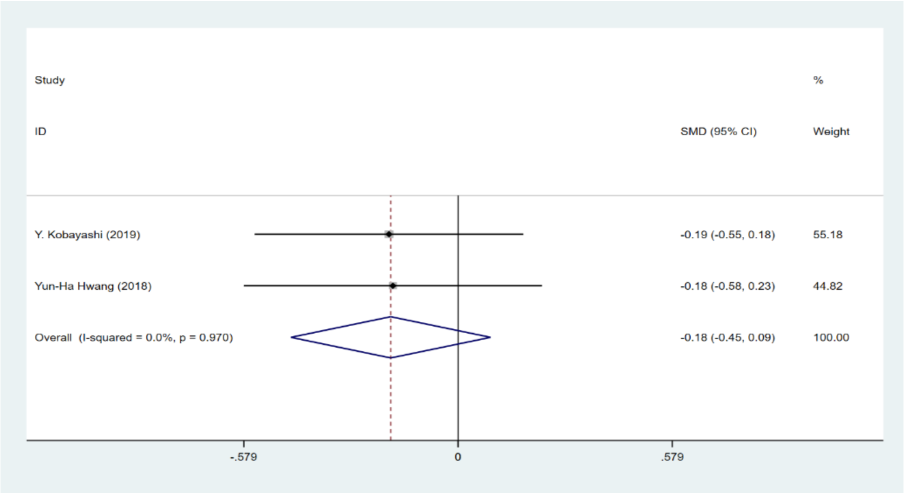

AST (MCI)

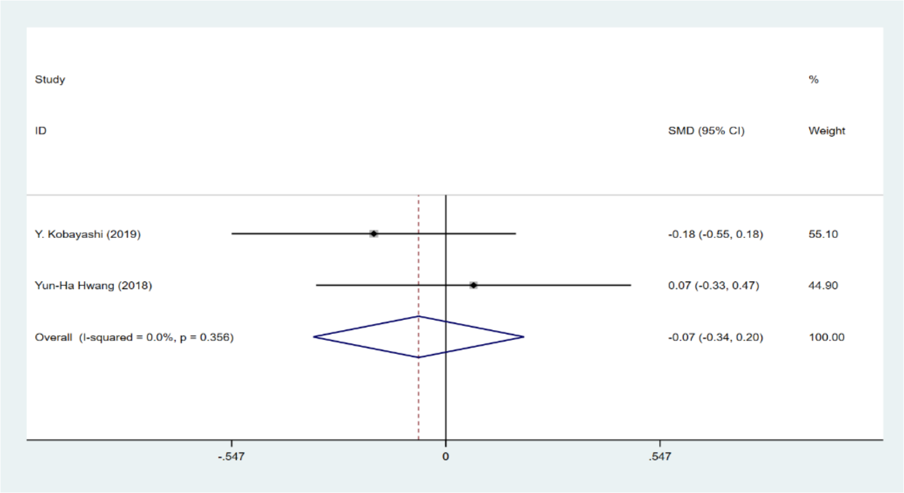

BUN (MCI)

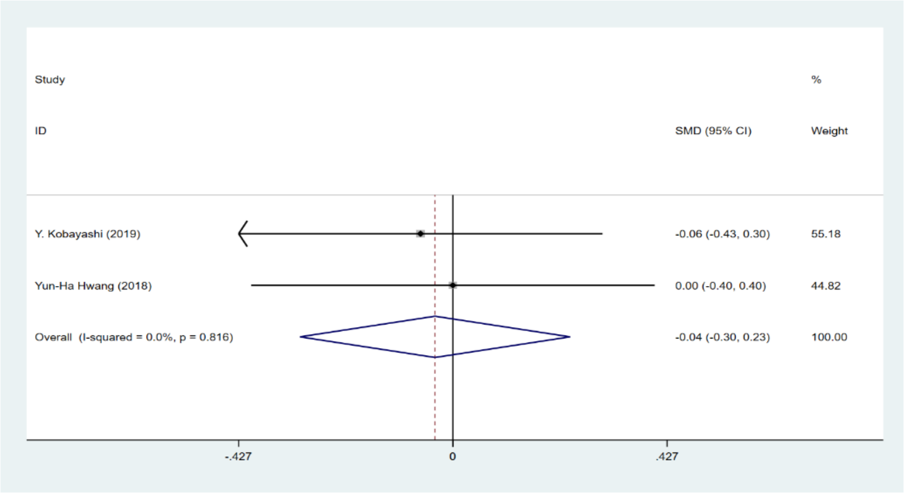

Hb (MCI)

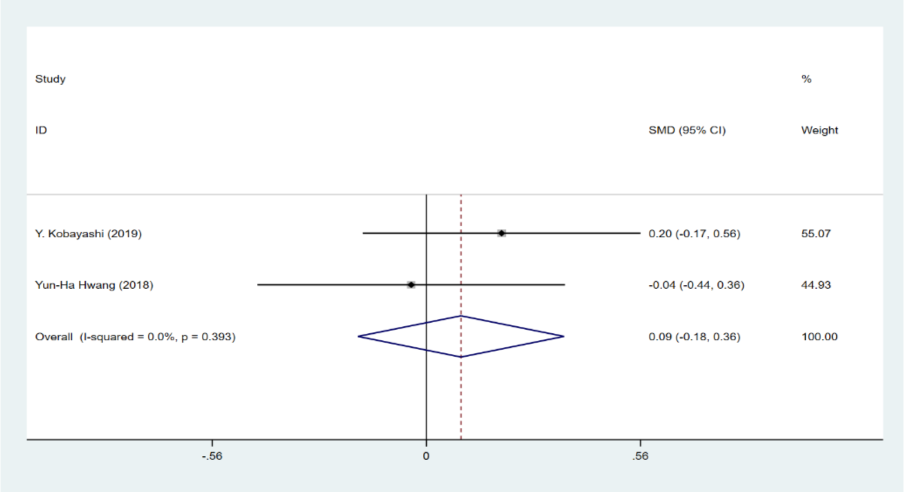

Glucose (MCI)

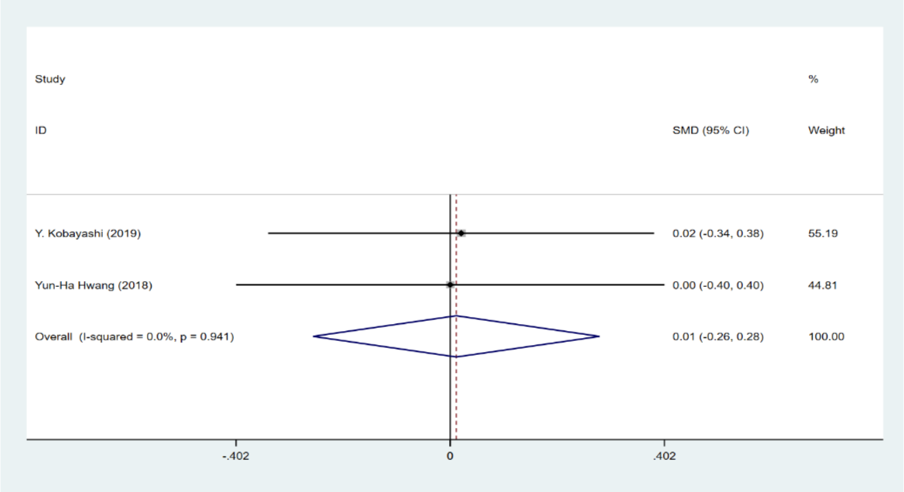

Ht (MCI)

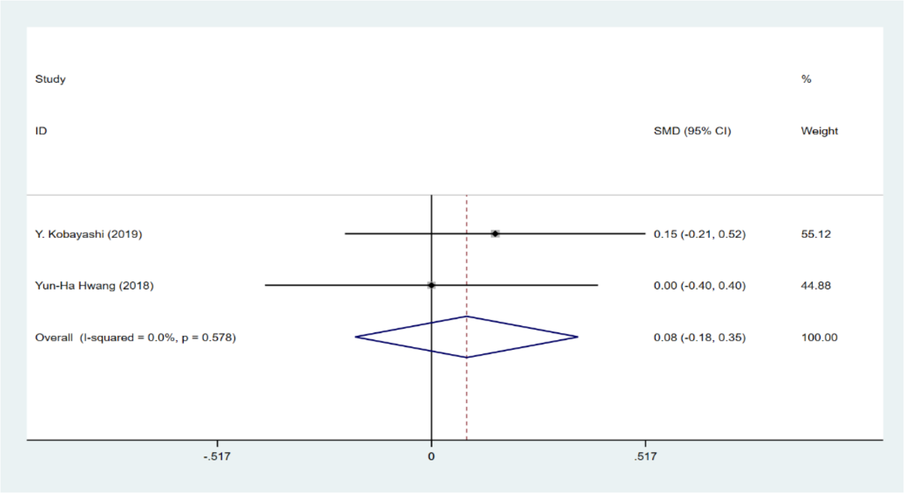

Platelet (MCI)

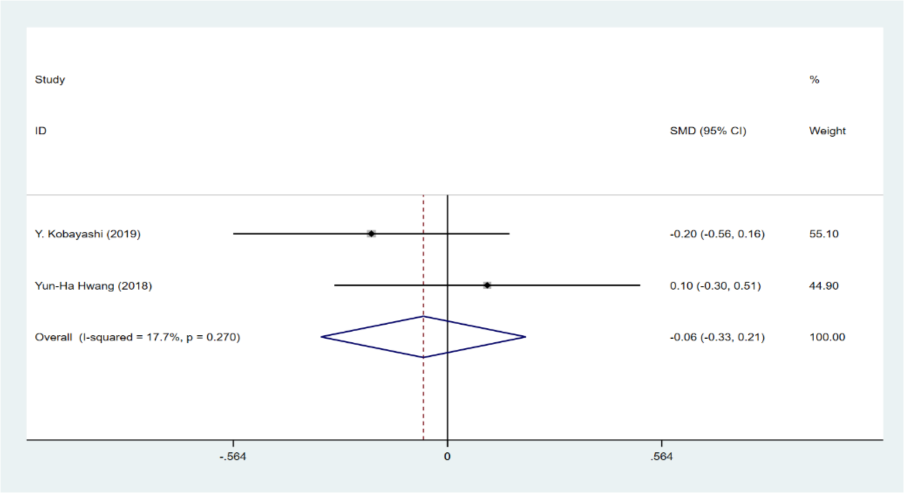

TBil (MCI)

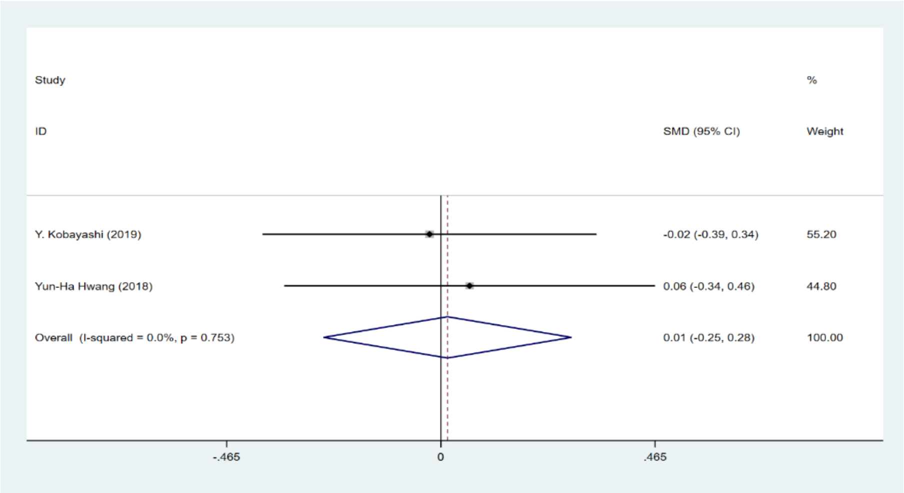

RBC (MCI)

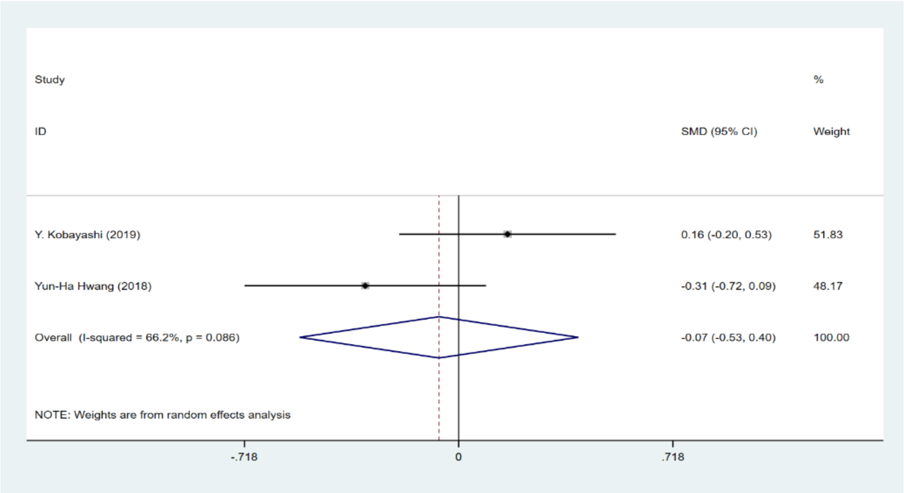

TP (MCI)

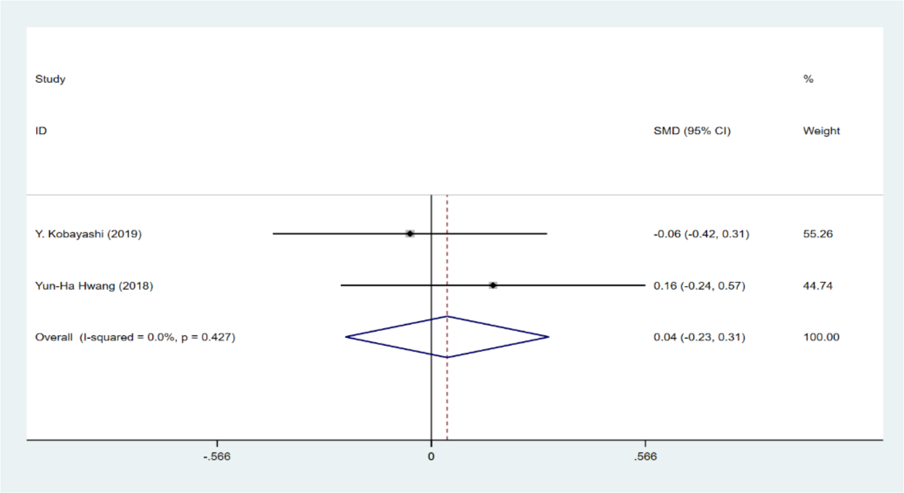

UA (MCI)

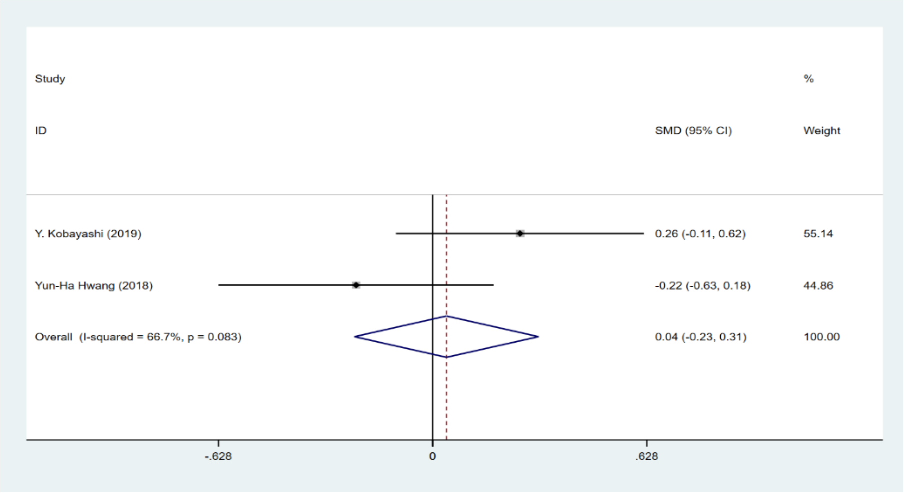

DBP (MCI)

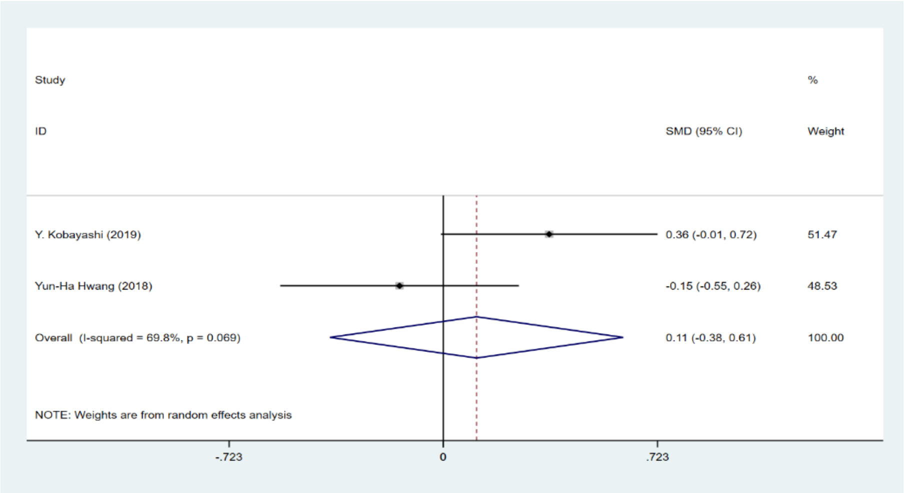

WBC (MCI)

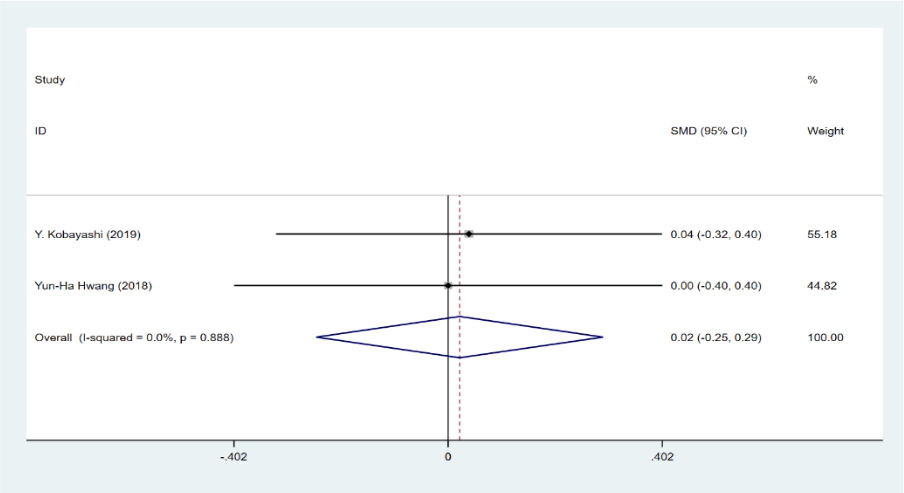

SBP (MCI)

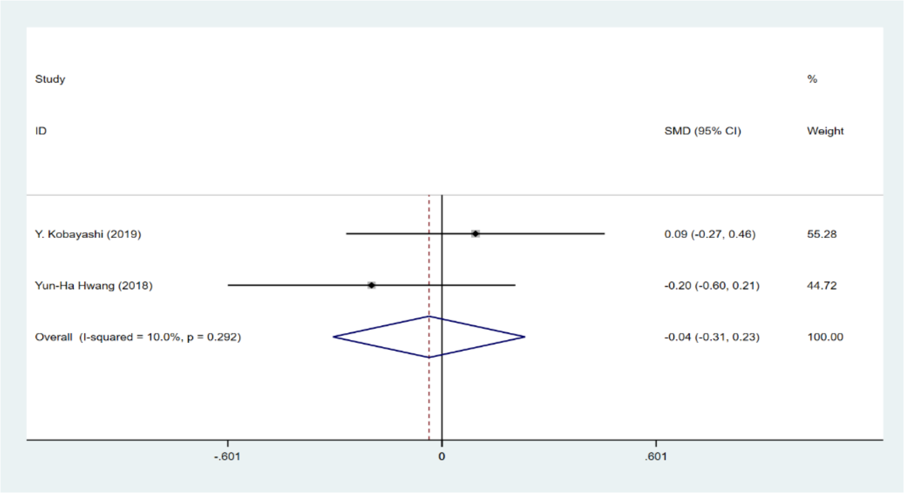

HR (MCI)

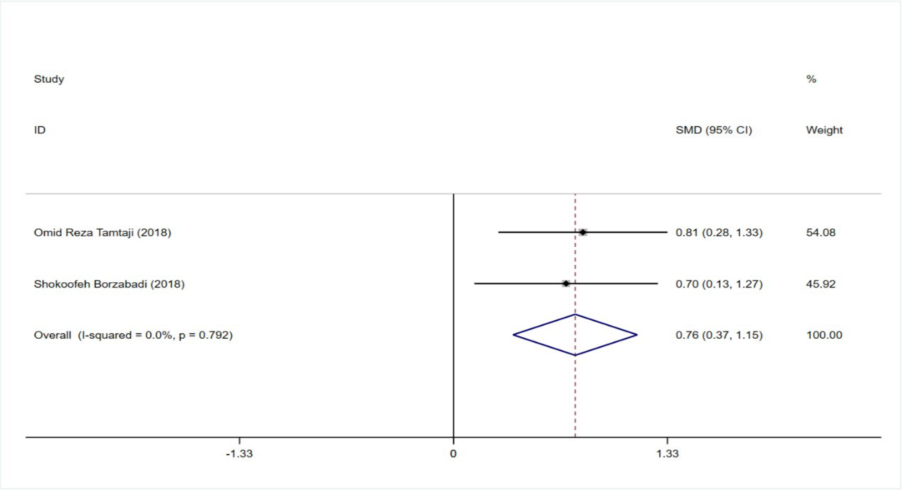

GSH (PD)

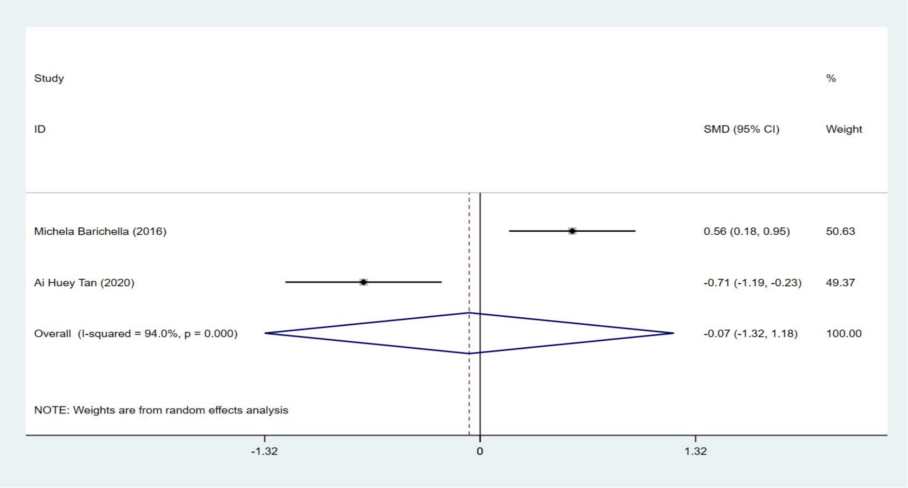

fecal viscosity (PD)
